# An integrated model for pre- and post-harvest aflatoxin contamination in maize

**DOI:** 10.1038/s41538-023-00238-7

**Published:** 2023-11-18

**Authors:** Richard O.J.H. Stutt, Matthew D. Castle, Peter Markwell, Robert Baker, Christopher A. Gilligan

**Affiliations:** 1 Epidemiology and Modelling Group, Department of Plant Sciences, University of Cambridge, Downing Street, Cambridge, CB2 3EA UK; 2 Cambridge Centre for Data-Driven Discovery, Department of Genetics, University of Cambridge, Downing Street, Cambridge, CB2 3EH UK; 3 Mars Global Food Safety Center, Mars Inc., Yanqi Economic Development Zone, Huairou, Beijing China

**Keywords:** Complexity, Risk factors

## Abstract

Aflatoxin contamination caused by colonization of maize by *Aspergillus flavus* continues to pose a major human and livestock health hazard in the food chain. Increasing attention has been focused on the development of models to predict risk and to identify effective intervention strategies. Most risk prediction models have focused on elucidating weather and site variables on the pre-harvest dynamics of *A. flavus* growth and aflatoxin production. However fungal growth and toxin accumulation continue to occur after harvest, especially in countries where storage conditions are limited by logistical and cost constraints. In this paper, building on previous work, we introduce and test an integrated meteorology-driven epidemiological model that covers the entire supply chain from planting to delivery. We parameterise the model using approximate Bayesian computation with monthly time-series data over six years for contamination levels of aflatoxin in daily shipments received from up to three sourcing regions at a high-volume maize processing plant in South Central India. The time series for aflatoxin levels from the parameterised model successfully replicated the overall profile, scale and variance of the historical aflatoxin datasets used for fitting and validation. We use the model to illustrate the dynamics of *A. flavus* growth and aflatoxin production during the pre- and post-harvest phases in different sourcing regions, in short-term predictions to inform decision making about sourcing supplies and to compare intervention strategies to reduce the risks of aflatoxin contamination.

## Introduction

A recent analysis of European Food Safety Agency (EFSA) data reveals that up to 80% of the crops grown, stored and traded worldwide are contaminated with detectable quantities of secondary fungal metabolites classed as mycotoxins, with as many as 20% over legal limits for contamination^1^. Among mycotoxins, aflatoxin B_1_ (AFB_1_) is one of the most potent human carcinogens^2^. Aflatoxin poisoning can occur directly, via skin contact with contaminated field crops and stored produce, but more commonly through ingestion, either of contaminated crop products or secondary products, such as milk, from animals that have consumed contaminated feed. Aflatoxin poisoning is implicated in delayed development in children and severe liver damage resulting in liver cancer^3^. *Aspergillus flavus* is a major source of AFB_1_. It is a widely distributed, prolific soil saprotroph that is also capable of infecting a wide range of crops, including cereals, legumes and tree nuts.

Here we focus on aflatoxin risk to maize (*Zea may*s L.), where levels of mycotoxin contamination in this widely traded commodity are of increasing global concern. Specifically, we integrate meteorologically driven epidemiological models for pre- and post-harvest dynamics of *A. flavus* as a tool to predict, review and manage risks along the entire maize supply chain from farm to factory gate. We also introduce functions to simulate disease management scenarios including post-harvest drying and filtering.

Maize is extensively cultivated around the world, with an annual global production exceeding one billion metric tons, covering 200 million hectares (García-Lara & Serna-Saldivar, 2019^4^). While up to 85% is traded for livestock feed, industrial products and biofuels among developed economies^5^, it remains the primary income source and an important component for nutrition in the diets of many people in countries across Sub-Saharan Africa, Latin America, and Asia^6,7^. Moreover, producers and consumers from low- and middle-income countries in tropical and sub-tropical regions are most at risk to mycotoxin exposure^8^. Climatic conditions are optimal for the development of aflatoxins in these regions and infrastructure and access to new technologies for storing, transporting and processing grain are often lacking.

Maize is susceptible to infection and colonisation by *A. flavus* and aflatoxin production during the pre-harvest and post-harvest phases of crop growth and storage. Spores of *A. flavus*, in the form of wind-dispersed conidia released from mycelium and sclerotia on soil surfaces, infect the developing inflorescences of maize. The fungus invades the grain, producing aflatoxin. Fungal growth and aflatoxin production are strongly influenced throughout the pre- and post-harvest phases by ambient temperature and moisture availability^9,10^.

**Fig. 1:**
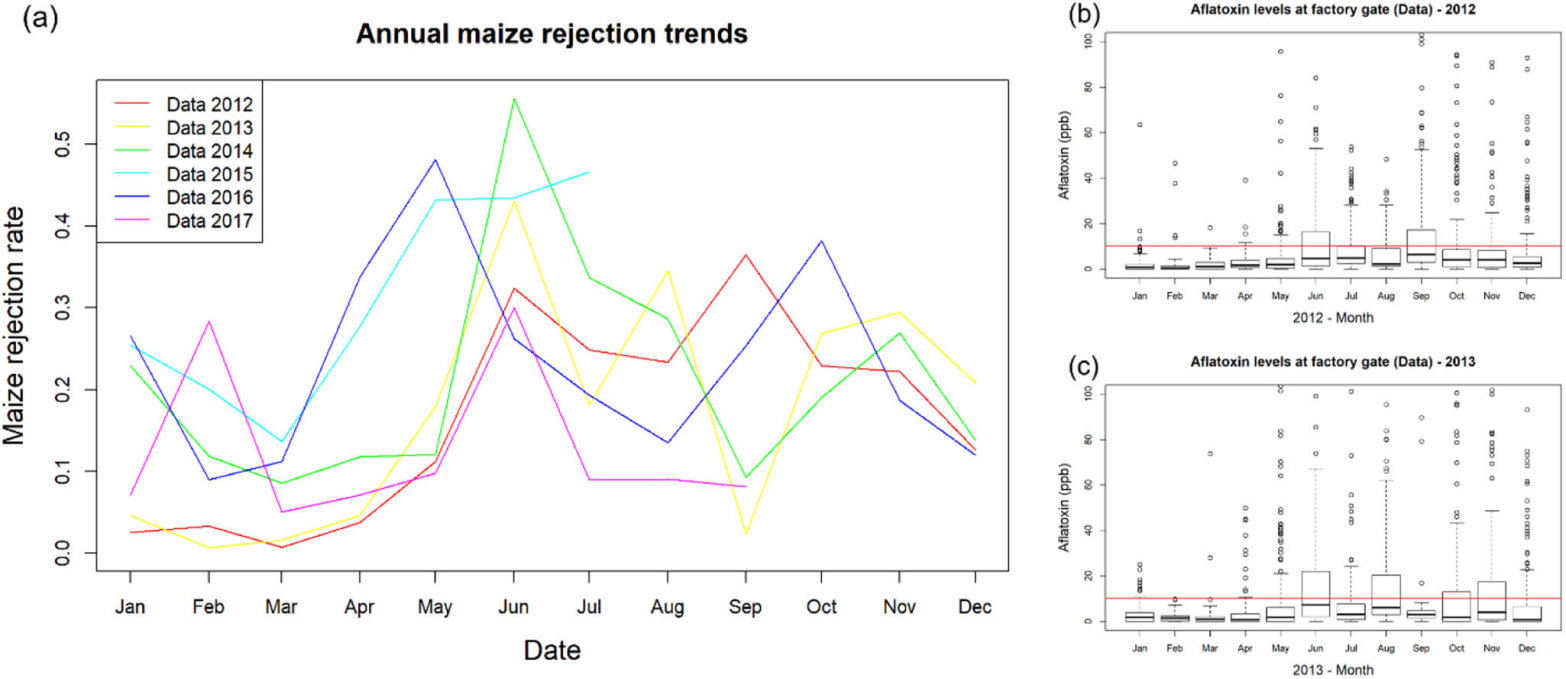
Motivating data. **a** Monthly rejection rates at MARS processing facility due to sampled aflatoxin levels exceeding 10-ppb limit for 2012–2017 and monthly sampled aflatoxin distributions for 2012 (**b**) and 2013 (**c**). In the boxplots (**b**) and (**c**) the bold black line indicates the median monthly sampled aflatoxin concentrations; the edges of the box are the 25th and 75th percentiles of monthly aflatoxin concentration; the whiskers show the range of datapoints within 2.5 times the interquartile range from the box, with points beyond this range are shown as individual circles.

The current work was motivated by a practical problem concerning high levels of rejection of shipments of maize due to aflatoxin at a high-volume maize processing plant in Hyderabad in India. In the region of interest at the time of the study, maize was grown predominantly (~80%) by smallholder farmers, with production being aggregated at regional markets for storage and later sale to consumers. The shipments were primarily rejected for exceeding the EU limit of 10 ppb for aflatoxin B1 in feed products^11^ (Annex I Section II: Mycotoxins). The processing plant was routinely rejecting 20% of monthly shipments rising to 40–55% in some years (Fig. 1). The high rejection rate underlined the need for a model to analyse and predict risk of contamination.

Most quantitative and modelling approaches to assessing the risks of aflatoxin have focused on modelling within field pre-harvest dynamics^12–18^. It is widely recognised, however, that *A. flavus* growth and AFB_1_ contamination continue throughout prolonged periods of grain storage after harvest^19^. An initial audit for India indicated a strong locational effect of where maize was grown and then stored, often at multiple sites in the supply chain, on subsequent levels of aflatoxin contamination. The dual cropping seasons for Kharif and Rabi crops also affect the duration and impact of storage on subsequent contamination. Accordingly, in this paper, we develop and test a model that integrates pre- and post-harvest dynamics of *A. flavus* and production of AFB_1_ to assess risk of contamination. Building on the work of Battilani et al.^12^. We produce a meteorologically-driven, spatially-structured post-harvest model that is coupled with a meteorologically-driven spatially-structured pre-harvest model that allows us to follow batches of maize through the supply chain. Our integrated model takes account of dual cropping seasons and multiple (three) sourcing regions for maize crops within a supply chain in India and is validated using data available from the maize processing plant in Hyderabad.

The primary aim is to predict the level of aflatoxin seen at the factory gate via a model of where and when the crop is grown, harvested and stored with a view to optimising sourcing, and to produce a framework that is adaptable for different countries and meteorological environments. Highly spatially resolved pre- and post-harvest data are scarce especially in low- and middle-income countries. We therefore use reliable data for AFB_1_ contamination on shipments reaching the factory gate for processing to parameterise and validate the model. We assess the performance of the model in simulating and predicting timeseries for *A. flavus* contamination and aflatoxin levels in batches from different sourcing regions during the cropping and storage phases of the supply chain. Following parameterisation and validation, we illustrate the use of the model as a tool for nowcasting (i.e. short-term prediction of unknown current and near-future status) for decision support and for scenario analysis to assess the effectiveness of different intervention and sourcing strategies in minimising the risk of aflatoxin contamination.

## Methods

### Meteorological data

Meteorological data for temperature, humidity and rainfall in the target regions were obtained from the UK Met Office’s Unified Model^20^ for the years corresponding to the aflatoxin time series data used for model training (2012–2015) and validation (2016–2017). The meteorological data are provided with 3-hourly temporal and 10 km resolution. The temporal data were linearly interpolated to 1-hourly temporal resolution.

### Maize supply data for model parameterisation and validation

Data for aflatoxin concentration for daily shipments arriving at a maize processing facility in Hyderabad, India operated by MARS Inc were used for parameterization and validation. Batches of maize are taken from storage in markets and sent as shipments to the factory on a daily basis throughout the year. The maize is shipped in 50–60 kg jute bags^21^ on trucks holding a mean of five tons of maize, with an average of seven shipments delivered per day. The maize is obtained from different commercial suppliers who source their maize from distinct sourcing regions, Bellary Guntur and Nizamabad, within Karnataka, Andhra Pradesh and Telangana States, respectively, at different times of the year. Upon receipt of a shipment of maize at the processing facility, factory staff test samples of each shipment for aflatoxin content, recording total aflatoxin concentration in ppb for all types combined (B1, B2, G1 and G2) for each shipment. Aflatoxin time series data for 2012–2015 recorded at the processing factory were used to fit and parameterize the model. Aflatoxin time series data for 2016–2017 were used for additional validation in which model predicted aflatoxin concentration and monthly shipment rejection rates are compared with historical data.

Maize is grown in India during two distinct (Rabi and Kharif) growing seasons, generally on smallholder farms. Rabi crops are planted between October and December and harvested between March and May, whereas Kharif crops are planted between June and August and harvested between November and January. After harvesting and de-cobbing, maize kernels may be subject to processing such as drying or filtering. After a short period of on-farm storage the maize kernels are taken to local markets (Mandis) within each region in 50–60 kg jute bags. Here the individual bags of maize are bought and sold in batches, before being either sent on immediately to a final destination (the Mars maize processing factory in the present case) or being stored locally in warehouses (some of which are climate controlled) until required. At each stage in the life history of a single batch of maize (whilst on the plant in the field, or within a bag in transit and storage), local environmental conditions, notably temperature, humidity and rainfall, affect the biological processes that govern *A. flavus* growth rates and aflatoxin production rates.

### Mechanistic modelling

**Fig. 2:**
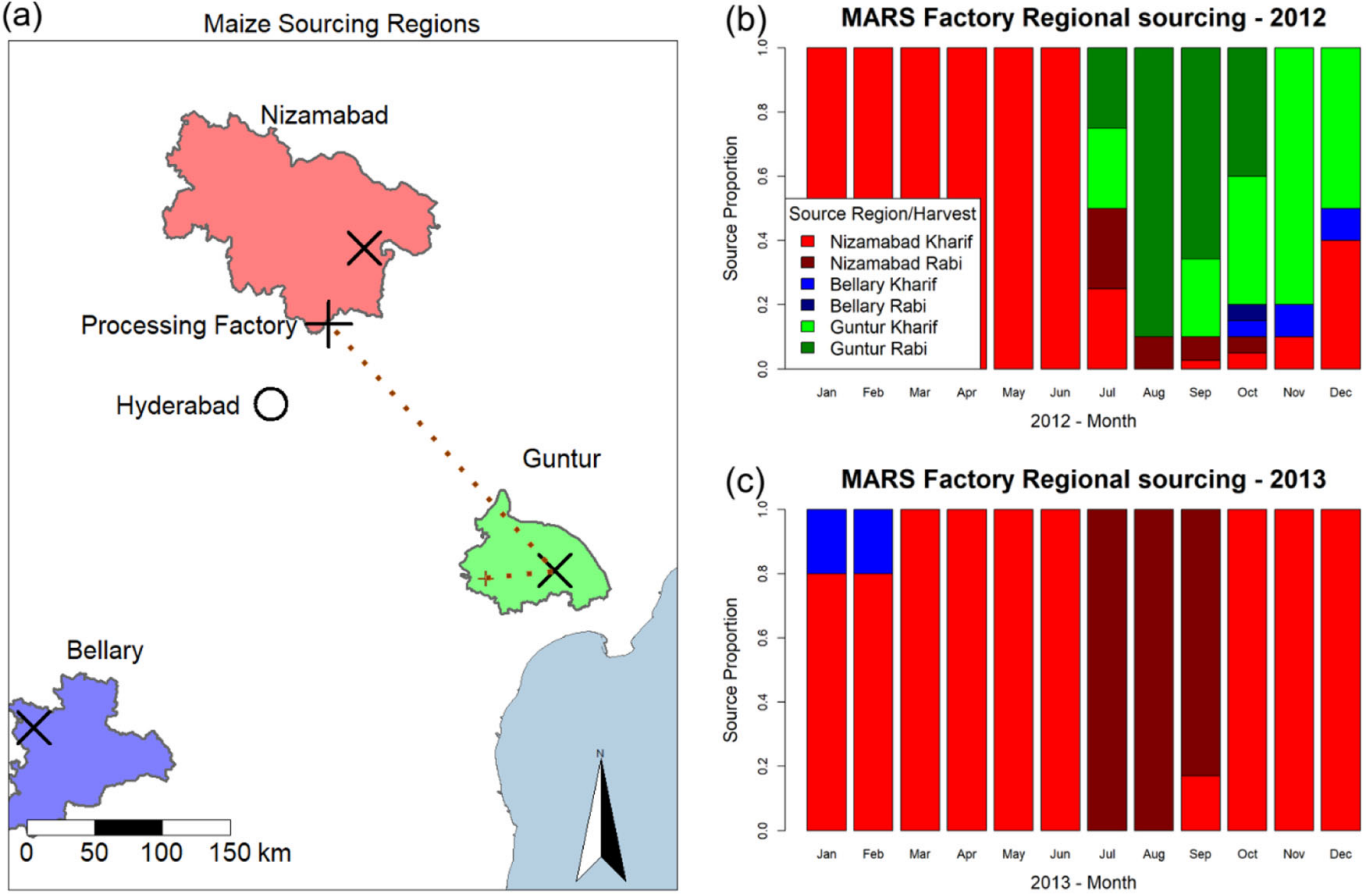
Regional map. **a** Map of the region of India used for sourcing by the processing factory (+), including the catchment areas and locations of the markets (x) in the Nizamabad, Guntur and Bellary areas. The major city of Hyderabad (o) is located to the south of Nizamabad. An example of the life history of a batch is indicated by the dotted line. The batch is initially planted in Guntur at the location marked with a beige x. The batch is later harvested and moves to the Guntur market. Finally, after some time in storage the batch is moved to the processing factory. Monthly profiles showing factory sourcing as proportion of shipments originating from each region and harvest for 2012 (**b**) and 2013 (**c**), See Supplementary Fig. 3 for full historical sourcing information.

We use a discrete-time compartment model to track *A. flavus* and aflatoxin levels on maize within the pre-harvest, processing and post-harvest components of an integrated model. The pre-harvest model tracks the colonisation and growth of *A. flavus* and aflatoxin accumulation in Kharif and Rabi maize crops on one thousand simulated farms in each of the three representative sourcing regions each season. After a harvest processing stage, the post-harvest model then tracks the growth of *A. flavus* and aflatoxin accumulation on the harvested grain on-farm and in-store, allowing for the influence of cultural practices to reduce infection as well as movement and storage of batches of maize in the sourcing regions before arrival at the factory-gate in Hyderabad. Hourly resolution meteorological data are used to drive growth and susceptibility of maize, the growth of *A. flavus* and accumulation of aflatoxin on farm and in store (Fig. 2).

A rectangular region (longitude 76.67° to 81.42° and latitude 14.20° to 19.70°) was chosen to cover the relevant sourcing areas and the processing site. This region was divided into a grid of 3,762 “cells” (57 × 66), each of which was 1/12 × 1/12 decimal degree, approximating to 10 km × 10 km in size.

Temperature, rainfall and relative humidity data were extracted for the target regions in each cell at 3-hourly temporal resolution from January 2011 to September 2017. The temporal data were linearly temporally interpolated to 1-hourly resolution resulting in ~180,000 spatially explicit maps of the ambient environmental conditions from which hourly maps were constructed as driving variables for sporulation, liberation and germination rates of *A. flavus*, and for relative growth and relative aflatoxin production rates. Mapping the three supply regions onto the spatial grid gives 383 meteorologically unique cells where maize could be grown: Nizamabad (223), Bellary (95) and Guntur (65). Each season, we seed 1000 fields with a random location and sowing date in each of the three regions for a total of 6000 simulated fields seeded per year. The fields are simulated on an hourly timestep through to a per-field harvest date at which point the material is processed and then moved to storage. Shipments in storage follow the dynamics of the hourly post-harvest model until such time as they are either selected for sourcing or discarded if not sourced after a year in storage. Shipments which have reached storage are available for sourcing, and a random weighted sample of ten of these are delivered to the factory each day according to the regional sourcing profile for that month.

#### Pre-harvest model

Using the models described below we simulate maize growth and *A. flavus* dynamics in individual fields to generate a distribution of pre-harvest *A. flavus* levels for each source region. In the absence of detailed information on exact sowing and harvesting dates for the Rabi and Kharif crops we assumed a uniform distribution of sowing dates: 16th October to 30th November for Rabi and 1st June and the 31st of July for Kharif. When initially planted, crops are introduced as free of *A. flavus*, aflatoxin and spores. Maize crops are harvested after an individual field accumulates 1500 growing degree days, at which point the batch moves to the harvest processing stage of the model.

**Fig. 3:**
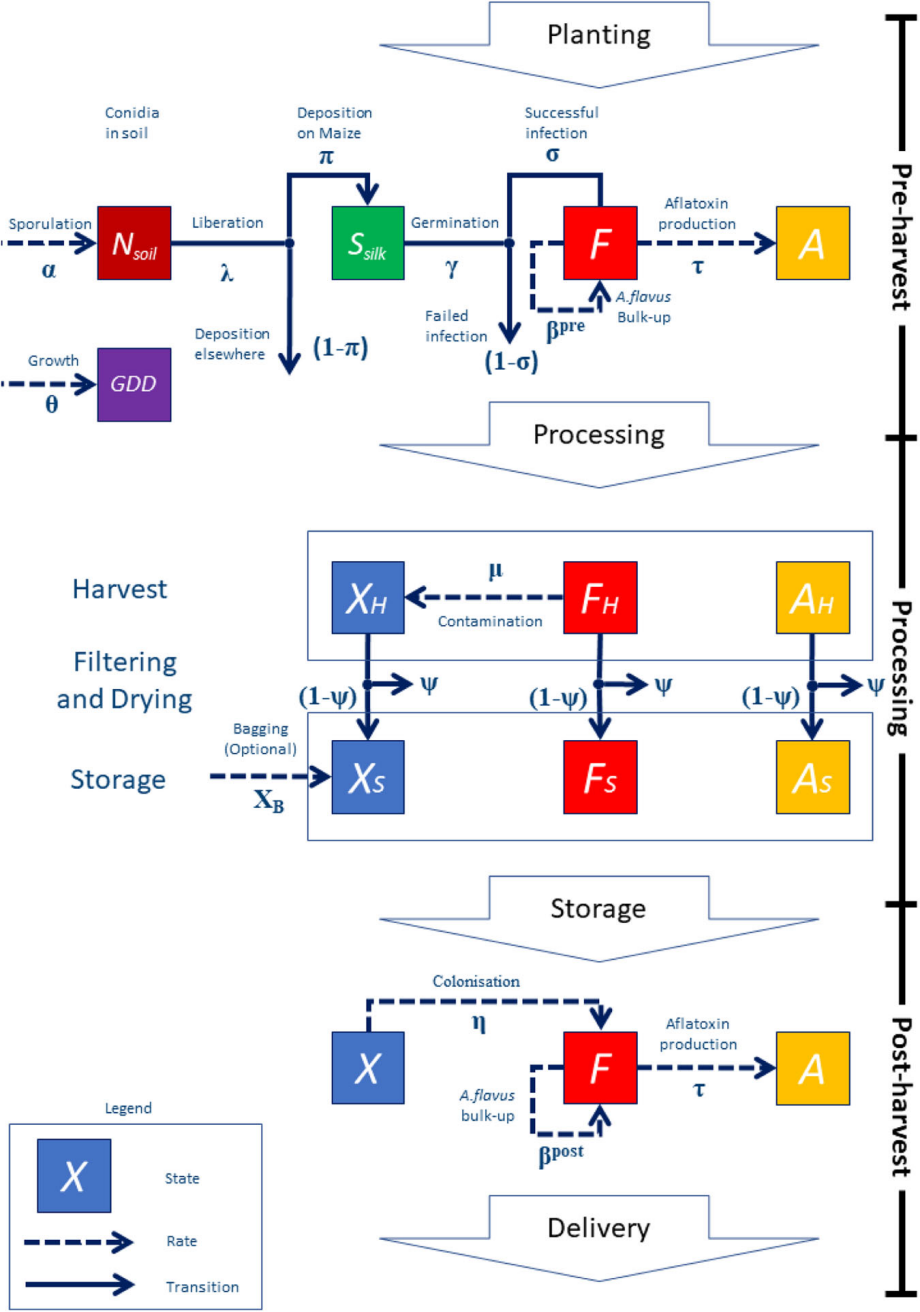
Model schematic. Schematic diagram describing the pre-harvest, processing and post-harvest integrated model for *A. flavus* growth and aflatoxin production on a postulated batch of potential maize kernels in the supply chain: N_soil_ represents the number of spores in the soil; S_silk_ represents the number of spores on the silks of the maize plants. The F compartment represents the level of *A. flavus* infection within the maize kernels. The A compartment represents the amount of aflatoxin within the kernels. X represents the amount of *A. flavus* present on contaminant material (fines) within bags. Subscripts H and S denote the values of the state variables at the time of harvest and entering storage, respectively. The key parameters are summarised in Table 1. (See Supplementary Table 1 for full description of functions controlling dynamic changes). All parameters and rates vary as appropriate depending on the hourly meteorological data at the current location of the maize shipment.

The pre-harvest model builds on Battilani et al.^12^ and adapts a modified version of their algorithm using an explicit epidemiological compartmental framework (Eq. 1) for the level of *A. flavus* infection and aflatoxin production within a maize crop (Fig. 3). The pre-harvest epidemiological model is described by discrete time equations for five state variables (with dimensions of unit area of crop). The state variables and parameters are updated hourly for each separate simulated field, with time *t* + 1 representing the state one hour after time *t*.

1 $$\begin{array}{cccc}A.{flavus}{\rm{spores\; in\; soil}} & {N}_{t+1} & = & {N}_{t}+{\alpha }_{t}-{\lambda }_{t}{N}_{t}\\ A.{flavus}{\rm{spores\; on\; silks}} & {{\rm{S}}}_{t+1} & = & {{\rm{S}}}_{t}+{{\rm{\pi }}}_{t}{{\rm{\lambda }}}_{t}{N}_{{\rm{t}}}-{\gamma }_{t}{S}_{t}\\ A.{flavus}{\rm{infections\; on\; maize}} & {F}_{t+1} & = & {\beta }_{t}^{{pre}}{F}_{t}\left(1-{F}_{t}\right)+{\sigma }_{t}{\gamma }_{t}{S}_{t}\\ {\rm{Aflatoxin\; on\; maize}} & {A}_{t+1} & = & {A}_{t}+{\tau }_{t}{F}_{t}\\ {\rm{Growing\; degree\; days}} & {{GDD}}_{t+1} & = & {{GDD}}_{t}+{\theta }_{t}\end{array}$$

The principal parameters are listed in Table 1: see also Supplementary Table 1 for a complete summary of the functions used in the models. The pre-harvest model uses results from Battilani et al.^12^, Giorni et al.^22^ and Kruit et al.^23^ to describe meteorologically-driven sporulation and infection processes (e.g. hourly sporulation rate as a function of local air temperature and relative humidity). We follow Shaykewich^24^ and Yan & Hunt^25^ in modelling maize growth using an accumulated thermal unit (growing degree day, GDD) process, by calculating GDD contributions from hourly temperature data. Internal water activity, *aw*_i_, is determined by the growth stage of the plant (GDD). This, in turn, allows us to model maize susceptibility using results from Siriacha^26^. These sources of information allow us to calculate infection process rates, *A. flavus* growth rates and susceptibility rates at an hourly resolution using the local environmental data for the target regions.

**Table 1:** Summary of key parameters used in the integrated model for pre-harvest, processing and post-harvest dynamics of *A. flavus* growth and aflatoxin production.

Variable/ Parameter	Description	Rate constant
Pre-harvest model: *A. flavus* growth and aflatoxin production		
α(T, H)	Sporulation rate	α_0_ (Estimated) = 1.00 × 10^−5^
λ(T, H)	Liberation rate	λ_0_ = 1.0^a^
π(T, H)	Deposition proportion	π_0_ = 1.0^a^
γ(T, H)	Germination rate	γ_0_ = 1.0^a^
σ(GDD)	Susceptibility	σ_0_ = 1.0
${\beta }^{{pre}}$ (T, H)	Pre-harvest *A. flavus* growth rate	${\beta }_{0}^{{pre}}$ (Estimated) = 6.31 × 10^−3^
τ(T, H)	Aflatoxin production rate	τ_0_ (Estimated) = 5.06
θ(T)	Thermal time	Θ_0_ = 1
Post-harvest model: Processing		
μ	Contaminant rate	μ = 0^b^
ψ	Filtering efficacy for removal of contaminants	ψ = 0^b^
δ	Drying protection period	δ (Estimated) = 25.0
X_B_	Bagging contamination rate	X_B_ = 0^b^
Post-harvest model: *A. flavus* growth and aflatoxin production		
η(T, H)	Contaminant colonisation rate	η_0_ = ${\beta }_{0}^{{post}}$
${\beta }^{{post}}$ (T, H)	Post-harvest *A. flavus* growth rate	${\beta }_{0}^{{post}}$ (Estimated) = 1.12 × 10^−3^
τ(T, H)	Aflatoxin production rate	τ_0_ (Estimated) = 5.06

Note that the rate constants λ_0_, π_0_, γ_0_ and σ_0_ are all set to 1.0 without loss of generality as these values are absorbed into the fitted constant α_0,_ while parameters μ, ψ and X_B_ were set to zero during parameter estimation. Estimated parameters presented to three significant figures. All parameters are driven by the hourly meteorological data at the current location of the maize shipment, where T = T(t, x) and H = H(t, x) represent the hourly temperature and humidity at time t and location x. (See Supplementary Table 1 for full description of the non-linear functions controlling dynamic changes).

^a^Set to 1 without loss of generality because the parameters are subsumed into α_0._

^b^Set to zero during fitting process.

##### Estimated pre-harvest parameters

Three key biological parameters (the absolute aflatoxin production rate (τ_0_), the absolute pre-harvest *A. flavus* growth rate (${\beta }_{0}^{{pre}}$) and the absolute primary *A. flavus* sporulation rate (α_0_) (Table 1) could not be parameterised from pre-existing datasets and these three parameters were estimated by Approximate Bayesian Computation using the aflatoxin times series data.

#### Harvest processing model

The harvest processing model incorporates common cultural control practices. We include drying, filtering and bagging of maize kernels after harvest to improve flexibility for scenario testing of alternative control scenarios (Eq. 2, Fig. 3).

#### Filtering

At harvest, each batch of maize has an *A. flavus* (*F*_H_) and an aflatoxin (*A*_H_) level of contamination calculated from the pre-harvest model. Harvest and removal of maize cobs from plants and separation of the kernels leads to contamination of the kernels with small particles of potentially contaminated material (leaves, stems, dust) termed “fines”. Removal of these fines by filtering reduces mycotoxin contamination^27^. We introduce an additional state variable, *X*_H_, where ${X}_{{\rm{H}}}=\mu {F}_{{\rm{H}}},$ and the rate parameter μ (Table 1) is included in the parameter set for estimation from the time series data.

The efficacy of different filtering processes and machinery may vary, and the capacity of any given mechanism to remove fines, kernels infested with *A. flavus*, and kernels contaminated with aflatoxin is unknown (but could be parameterised given appropriate data). We incorporated filtering into the model framework through the inclusion of a parameter, ψ, corresponding to the filtering efficacy of the process on the amount of *A. flavus*, aflatoxin and fines. The process is described by the following equations:

2 $$\begin{array}{ccc}{F}_{S} & = & \left(1-\psi \right){F}_{H}\\ {A}_{S} & = & \left(1-\psi \right){A}_{H}\\ {X}_{S} & = & \left(1-\psi \right){X}_{H}\end{array}$$

where ${F}_{H}$ and ${F}_{S}$ correspond to the level of *A. flavus* in a batch before and after filtering.

#### Drying

Maize kernels are dried after harvest to reduce internal moisture content levels below the threshold at which *A. flavus* can both grow and produce aflatoxin^10,28,29^. Air drying by spreading maize kernels on the ground for exposure to the sun is common in low- and middle-income countries with mechanical drying in more intensive systems. In the absence of detailed information, the drying protection period (δ) was treated as a parameter to be estimated from time series data for aflatoxin contamination at the factory gate. We assume that drying interrupts fungal growth and toxin production, hence the *A. flavus* bulk-up and aflatoxin rates are set to zero in the model during estimated drying protection period. See Discussion for alternative approaches.

#### Bagging

In India, maize is generally stored in jute bags after harvesting and drying. The bags may be reused from season to season without effective cleaning, and so provide an additional source of inoculum at bagging time^21^. We incorporate contamination from bags by allowing an additional amount of fines (*X*_B_) to be added to the post-harvest contaminants (*X*_H_).

##### Estimated harvest processing parameters

For the purposes of fitting the integrated model to the time series data for aflatoxin levels at the factory gate (see below), we estimate the drying delay period (δ), but in the absence of additional information we treat the contaminant (μ), bag contamination (*X*_B_) and filtering (ψ) rates as fixed at zero (Table 1).

#### Post-harvest model

The post-harvest model extends from harvest to delivery to a factory, during which the material is in storage either on the original farm or at a market. The post-harvest model introduces the potential for controlled storage conditions, in which the environmental conditions are modulated with consequent effects on *A. flavus* and aflatoxin production. We assume that harvest processing precedes the start of storage. Each batch is therefore initially characterised by *A. flavus*, (*F*_S_), aflatoxin, (*A*_S_) and contaminant (*X*_S_) concentrations.

The post-harvest component of the model consists of two processes. Firstly, tracking *A. flavus* and aflatoxin levels in stored maize (using environmental weather data to drive the biological processes). Secondly simulating the sourcing and sampling process of maize at the factory gate in order to allow for matching of the model output to the available aflatoxin time series data.

The post-harvest epidemiological model is described by the following non-linear discrete time equations: (Eq. 3) parameters are described in Table 1 with further detail in Supplementary Table 1.

3 $$\begin{array}{ccc}{X}_{t+1} & = & {X}_{t}\\ {F}_{t+1} & = & {\beta }_{t}^{{post}}{(F}_{t}+{X}_{t})\left(1-{F}_{t}\right)\\ {A}_{t+1} & = & {A}_{t}+{\tau }_{t}{F}_{t}\end{array}$$

#### Colonisation and growth

Growth of *A. flavus* on the maize kernels occurs from *A. flavus* present on the kernels (*F*) or from other contaminant material (fines) within the bag (*X*). We assume a similar functional form for the *A. flavus* growth rate as for pre-harvest but with a different scaling parameter (${\beta }_{0}^{{post}}$). Here the water activity (see Supplementary Table 1 for details), is determined by ambient humidity levels, rather than the internal moisture content of the plant and is taken to be the maximum of the water activity due to humidity and dew point. See Supplementary Table 1 for details. The parameter ${\beta }_{0}^{{post}}$ is obtained by fitting the integrated model to time series data.

#### Aflatoxin production

The post-harvest aflatoxin production rate (τ) follows the form as for the pre-harvest model with water activity now related to ambient humidity levels. The rate constant τ_0_ is common between the pre- and post-harvest models, as the fitting process determined separate rate constants provided no significant benefit.

#### Storage and sourcing

The majority of maize in India is stored for several months, potentially up to a year, in non-climate-controlled spaces where it is exposed to ambient temperature and moisture conditions, which permit continued growth of *A. flavus* and aflatoxin production. However, some storage facilities are climate controlled. Controlled storage conditions can affect temperature, relative humidity and oxygen tension^22,30^. We therefore permit the model to adjust the temperature and humidity as specified for the storage where this is known. We allow for different storage conditions over the supply chain, with material stored on the farm and at market potentially having different storage conditions. In India, maize is typically stored on the farm for the first 30 days before being moved to the markets. The model reflects this, with batches being stored on the farm for the first 30 days after harvest, subject to the environmental conditions (and any controlled storage conditions) at that location before being moved to the market. Once arrived at the market, batches can be selected by the sourcing process to be sent as shipments to the factory.

#### Sourcing

Accurate simulation of aflatoxin levels in deliveries to the factory requires knowledge of the source of shipments. Data provided by Mars Inc for shipments received at the factory indicated the source market for a given shipment, but data were not available for the precise growing location of a given shipment. Hence, the shipment data were summarised on a monthly basis to determine the proportion of shipments originating from each growing region and harvest season (Kharif vs Rabi) (Supplementary Fig. 3). The summary sourcing data are used during the simulations, with shipments sourced from markets to the factory on a daily basis and the integrated models for *A. flavus* and aflatoxin production updated according to hourly meteorological data Each day of the simulation, shipments matching the required market and cropping season are selected randomly from the pool of available shipments at markets in proportion to the sourcing rates indicated in the historical data. See Supplementary Fig. 3 for full details of the sourcing data.

The pooled sourcing information constrains our fitting and validation comparing the distributions of aflatoxin values obtained over monthly periods values between historical data and model output, as while the hourly model generates a set of deliveries each day we lack the precise information to compare these simulated aflatoxin values to individual shipments.

##### Estimated post-harvest parameters

Two key biological parameters (the absolute aflatoxin production rate (τ_0_, a common parameter with the pre-harvest model), and the absolute post-harvest *A. flavus* growth rate (${\beta }_{0}^{{pre}}$) could not be parameterised from pre-existing datasets so were estimated by Approximate Bayesian Computation (ABC) using the aflatoxin times series data.

#### Simulated sampling to estimate aflatoxin levels at the factory gate

The aflatoxin time series data provided by MARS Inc. were derived from multiple samples from the same shipment. The high observed variance between successive samples from the same batch indicated the need to simulate the sampling process to capture this source of variability in order to make a fair comparison between model and historical observations. Hence, the model predicted aflatoxin values (*A*) were subjected to a simulated sampling process to obtain a value (*B*) for comparison with historical data by the following procedure:

$$B \sim \exp \left(\lambda =\frac{1}{A}\right)$$

where exp is the exponential distribution with mean $1/\lambda$.

### Parameter estimation

While some model parameters could be obtained from the literature (see Table 1 and Supplementary Table 1), five parameters were determined using ABC^31^. As there are no data available for intermediate stages in the supply chain, we therefore compared the model predictions with daily data recorded at the processing plant. Fitting was performed on data for 2012–2015, with 2016–2017 retained for validation. The key parameters to be estimated are primary *A. flavus* sporulation rate (α), pre-harvest *A. flavus* growth rate $({\beta }^{{pre}})$, post-harvest *A. flavus* growth rate $({\beta }^{{post}})$, aflatoxin production rate (τ) and drying protection duration (δ) (Table 1).

#### Overview of fitting algorithm

We sample a set of model parameters, η, independently from a constrained uniform prior distribution for each of the five parameters. The model is run over a given time range (2012–2015, the “fitting period”) with these parameters and a time series of daily delivery aflatoxin levels is generated. The model delivery aflatoxin time series is aggregated by month and the 75th percentile of the sampled aflatoxin levels are compared with the aggregated monthly observed data for aflatoxin levels using the following fitting metric, *E*.

$$E({\rm{\eta }})=\frac{1}{n}\sum _{\begin{array}{c}{months}\,i\\ {in}\,{fitting}\\ {period}\end{array}}{\left(\sqrt{{O}_{i}}-\sqrt{{M}_{i}({\rm{\eta }})}\right)}^{2}$$

Where ${O}_{i}$ and ${M}_{i}({\rm{\eta }})$ are the 75^th^ percentile of aflatoxin values for month *I* for the observed data and model predictions (given parameters η), respectively, and *n* is the number of months in the fitting period. Note that as the model is stochastic, multiple realisations with the same parameters (η) give different results, and thus a distribution of values for $E\left({\rm{\eta }}\right)$. The square root transformation was chosen for variance stabilising properties.

The posterior distribution was generated from 750,000 parameter samples, accepting the top 1% of parameter samples according to the fitting metric, and rejecting the remainder. The parameter space is then cut into 5-d boxes and the likelihood for parameter values within each box is calculated as the number of acceptances out of the total number of samples performed in that box. Given the uniform prior, this acceptance rate is then taken as the posterior probability distribution, shown in Supplementary Fig. 1. Further details are given in Supplementary Information section Model Parameter Estimation.

The values of epidemiological parameters selected from the posterior distribution for use in the model are recorded in Table 1.

#### Model validation

**Fig. 4:**
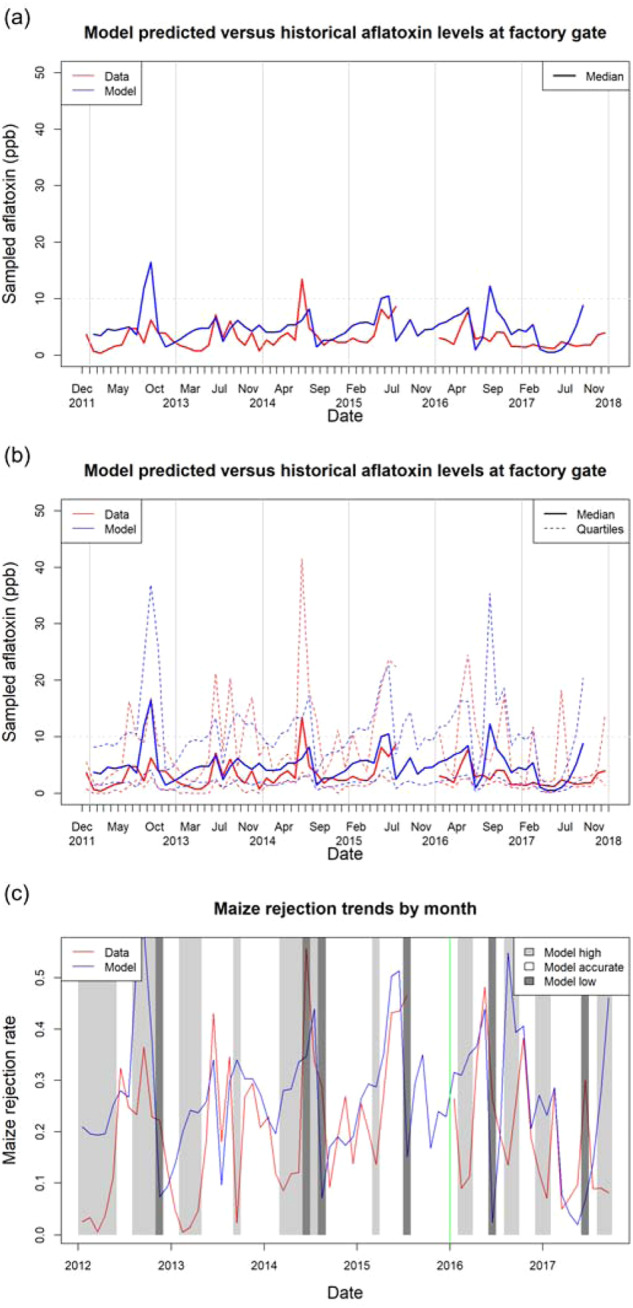
Comparison of model predicted and observed aflatoxin levels in shipments received at the maize processing plant in Hyderabad. Data were received from Kharif and Rabi crops grown in up to three sourcing regions (Bellary, Guntur and Nizamabad) during 2012–2017. The data from the hourly resolution model were pooled into monthly distributions of aflatoxin values, with (**a**) the median and (**b**) the 25th percentile, median and 75th percentile of the monthly aflatoxin values shown for the model and the median for historical observations. The 10-ppb aflatoxin rejection threshold is marked by a horizontal dashed line. **c** Comparison of model predicted and historically observed monthly rejection rates for shipments at the factory gate. The light grey shading indicates where the model is classified as too high (over 10% above the observed rejection rate) and the dark grey shading where the model is classified as too low (over 10% below the observed rejection rate). The divide between the periods used for fitting and validation data is denoted by the green vertical line. The hourly model generates a simulated life history of between one and eleven thousand hours from planting to delivery for each of the ~21,000 simulated shipments delivered in this time range.

The fitted model was validated against data for the years 2016–2017 (the “validation period”). We calculate descriptive statistics (monthly median aflatoxin concentration and monthly average shipment rejection rate) to characterise the model performance for the validation period relative to the fitting period. The parameterised model outputs for predicted monthly median aflatoxin concentration and shipment rejection rates are compared with the respective historical data. Each month the model predictions are classified as “Low”, “Accurate” or “High” depending on the performance of the model relative to the historic data. The “Accurate” classification criteria are model predicted monthly median aflatoxin concentration within $\pm 4{\rm{ppb}}$ of the historical monthly median aflatoxin concentration, and model predicted monthly rejection rate within $\pm 10 \%$ of historical monthly rejection rate. These classifications are performed for the fitting (2012–2015), validation (2016–2017) and entire (2012–2017) datasets. Numeric results are presented in Tables 2 and 3 as well as graphically in Fig. 4c.

**Table 2:** Descriptive statistics of model performance: median aflatoxin.

Dataset	Model performance		
	Low (<-4ppb)	Accurate ($\pm 4{\rm{ppb}})$	High (>+4ppb)
Fitting period (2012–2015)	4.7%	83.7%	11.6%
**Validation period (2016–2017)**	**0.0%**	**85.7%**	**14.3%**
Total period (2012–2017)	3.1%	84.4%	12.5%

Deliveries from the hourly model within each month classified as Low, Accurate, or High if the model predicted median aflatoxin levels is more than 4 ppb below, within 4 ppb of or more than 4 ppb above, respectively, relative to the historical observed median aflatoxin level. The table summarises the proportion of months with each classification for the respective dataset.

**Table 3:** Descriptive statistics of model performance: monthly rejection rates.

Dataset	Model performance		
	Low (<10%)	Accurate ($\pm 10 \% )$	High (>10%)
Fitting period (2012–2015)	9.3%	51.2%	39.5%
**Validation period (2016–2017)**	**9.5%**	**52.4%**	**38.1%**
Total period (2012–2017)	9.4%	51.6%	39.0%

Deliveries computed from the hourly model within each month classified as Low, Accurate, or High if the model predicted monthly rejection rate is more than 10% below, within 10% of or more than 10% above, respectively, relative to the historical observed monthly rejection rate. The table summarises the proportion of months with each classification for the respective dataset.

## Results

### ABC parameter estimation

Approximate Bayesian computation allowed estimation of five key epidemiological parameters for the integrated pre- and post-harvest model, using data for aflatoxin levels in batches arriving at the processing factory between 2012 and 2015 (Table 1; Supplementary Fig. 1). The fitting established plausible bounds on all parameters. The posterior distribution for the drying protection duration (δ) was particularly well defined within an estimated range of 10–35 days independent of other parameter values. There are trade-offs amongst certain parameter posterior distributions with correlations between the primary sporulation rate (α) and the pre-harvest bulk up rate (${\beta }^{{pre}}$) as well as the primary sporulation rate (α) and the toxin production rate (τ) (Supplementary Fig. 1). These correlations are consistent with the lack of observational data for *A. flavus* levels throughout the supply chain, other than for sourcing regions. Nevertheless, by sampling from the combined posterior distributions it is possible to predict final aflatoxin levels accurately.

### The integrated model for pre- and post-harvest dynamics

The integrated model replicated broad trends in the time-series for the median (Fig. 4a) and interquartile range (Fig. 4b) aflatoxin levels compared with the observed data, and the model correspondence to historical data is consistent across the fitting (pre-2016) and validation (2016–2017) time ranges. The model predicts the magnitudes and timings of annual peaks as well as monthly fluctuations in aflatoxin levels (Fig. 4a, b). The rejection rates predicted by the model are similar to the observed rejection rates and follow the same annual cycle of peaks and troughs (Fig. 4c). In terms of predicting the highest peaks in the data, the model is accurate with the highest peaks in 2013 and 2015, but overestimates in 2012 and underestimates in 2014. The distribution of model outcomes was also capable of replicating seasons with low median but high variability in aflatoxin levels such as 2016, one of the validation years (Fig. 4b: see also Supplementary Figs. 2, 4 for a detailed comparison of model outputs against historical observations including variance.) Also of note is that while there are discrepancies between the median of a single realisation of the model and the data median, the 95% credible interval for the model encompasses the median of the entire data set (see Supplementary Fig. 7).

The model performance in matching monthly median aflatoxin levels is summarised in Table 2. Monthly median aflatoxin levels for the model were classified as: Accurate if they were within $\pm 4{\rm{ppb}}$ of the observed monthly median aflatoxin concentration, Low if 4 ppb or more below, and High if 4 ppb or more above. The model performance was consistent across the data for the training period (2012–15) used for fitting and the validation period (2016–17), giving an accuracy of approximately 85% for being within $\pm 4{\rm{ppb}}$ of the validation data. The model is more likely to overpredict than underpredict median aflatoxin levels, indicating the model is less likely to underpredict a period of high risk (false negative) than to overestimate risk during low-risk times (false positive).

Predicted rejection rates were obtained by assessing the monthly proportion of aflatoxin values from model outputs that exceeded the 10-ppb threshold (Fig. 4c). The rejection rates follow the broad trends of historical rejection rates, capturing periods of high rejection rates, although the model does typically predict slightly higher rejection rates in periods that were historically low. The model performance in matching the monthly shipment rejection rates at the processing factory is summarised in Table 3. Monthly rejection rates for the model were classified as: Accurate if they were within $\pm 10 \%$ of the observed monthly rejection rate, Low if >10% below, and High if >10% above. The model performance was consistent across the data for the training period (2012–15) used for fitting and the validation period (2016–17), giving an accuracy of approximately 50% for being within $\pm 10 \%$ of the validation data. The model is significantly more likely to overpredict than underpredict rejection rates, indicating the model is much less likely to underpredict a period of high risk (false negative) than to overestimate risk during low-risk times (false positive). Overall, model overestimation of rejection rates is driven predominantly by periods when the historical rejection rates were low (Fig. 4b: see also Supplementary Fig. 5 for equivalent data aggregated to quarterly intervals and Supplementary Fig. 6 for a scatter plot comparison of monthly model and historical rejection rates.)

**Fig. 5:**
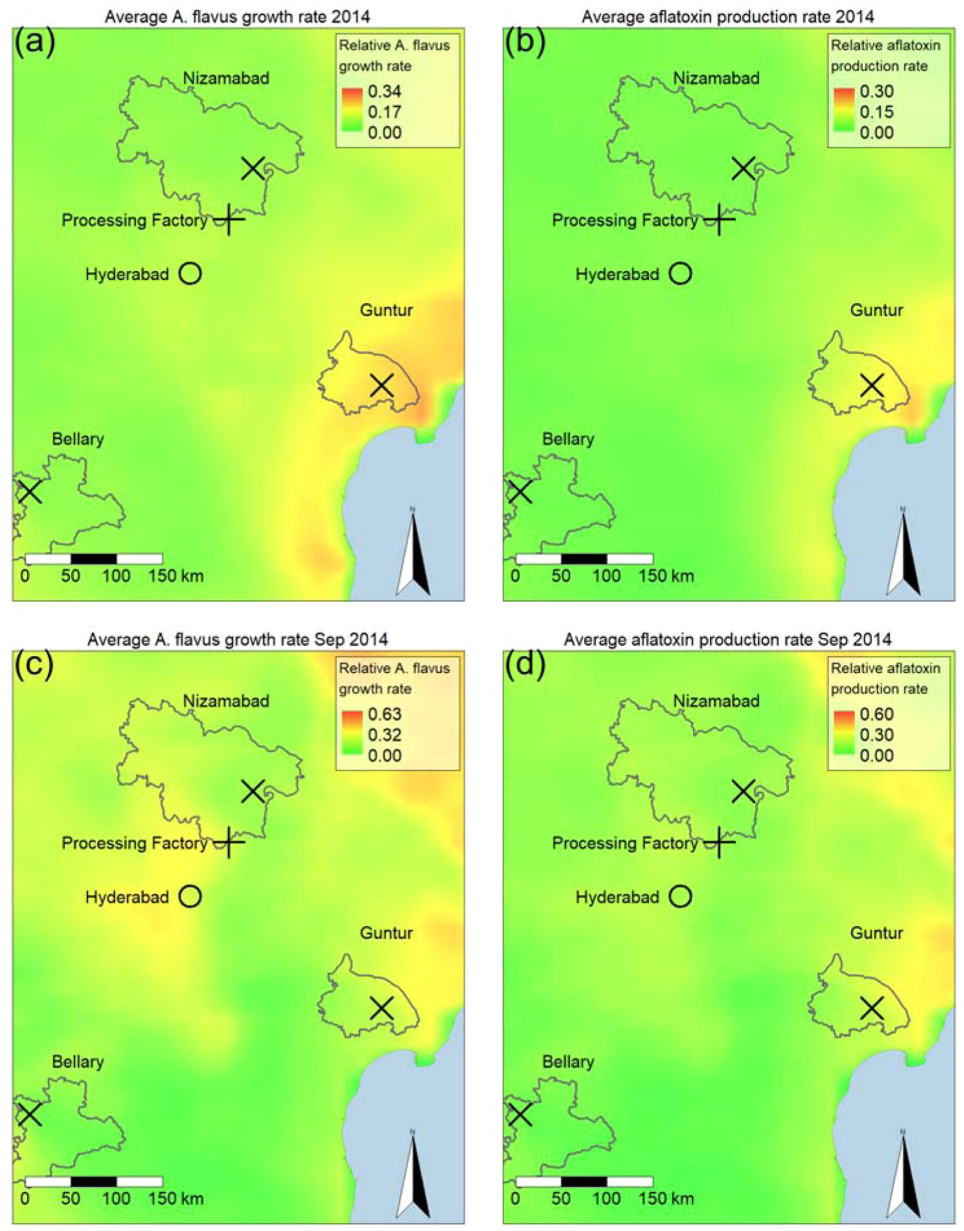
Maps for model predicted average hourly relative *A. flavus* growth rate (${\beta }^{{post}}/{\beta }_{0}^{{post}}$) and relative aflatoxin production rate (τ/${\tau }_{0}$) in the area covering the three sourcing regions in India in 2014. Panels (**a**) and (**b**) show the annual average of 8,760 hourly relative *A. flavus* growth and aflatoxin production rates for 2014, respectively. Monthly averages for 720 hourly relative *A. flavus* growth and aflatoxin production rates in September 2014 are shown in (**c**) and (**d**), respectively.

The spatial and temporal variability of the suitability for *A. flavus* growth and aflatoxin production in the region containing the sourcing regions for the Hyderabad processing plant is illustrated in Fig. 5. Note the different scales for the monthly and annual plots, with peak monthly average suitability being approximately twice that of peak annual average suitability. The spatial patterning of suitability areas of significantly higher risk on both annual and monthly timescales. The annual averages for 2014 highlight the coastal region near Guntur as a high-risk area for both *A. flavus* (Fig. 5a) and aflatoxin production (Fig. 5b). While there are spatial trends in suitability, there is significant variability in suitability over different time intervals and durations, with the possibility of periods of high risk over short time intervals in locations not identified from annual averages. In particular, September 2014, which had the highest monthly average *A. flavus* growth suitability (Fig. 5c) for that year, shows quite markedly different spatial patterning from the annual average (Fig. 5a). During September 2014, our results indicate the most suitable areas for *A. flavus* growth and aflatoxin production are inland, away from the coast, while the annual average suitability is mainly concentrated along the coast near Guntur. This variability highlights the necessity of considering both the location as well as the time at which maize is grown and stored when assessing aflatoxin risk. The areas suitable for aflatoxin production are typically a subset of those suitable for *A. flavus* growth, reflecting the assumption that conditions for aflatoxin production follow the same form as *A. flavus* growth with stricter constraints (Supplementary Table 1: see also Supplementary Fig. 9 for other years and months).

### Application of the model—*A. flavus* and aflatoxin profiles during the cropping and storage phases of supply chain

**Fig. 6:**
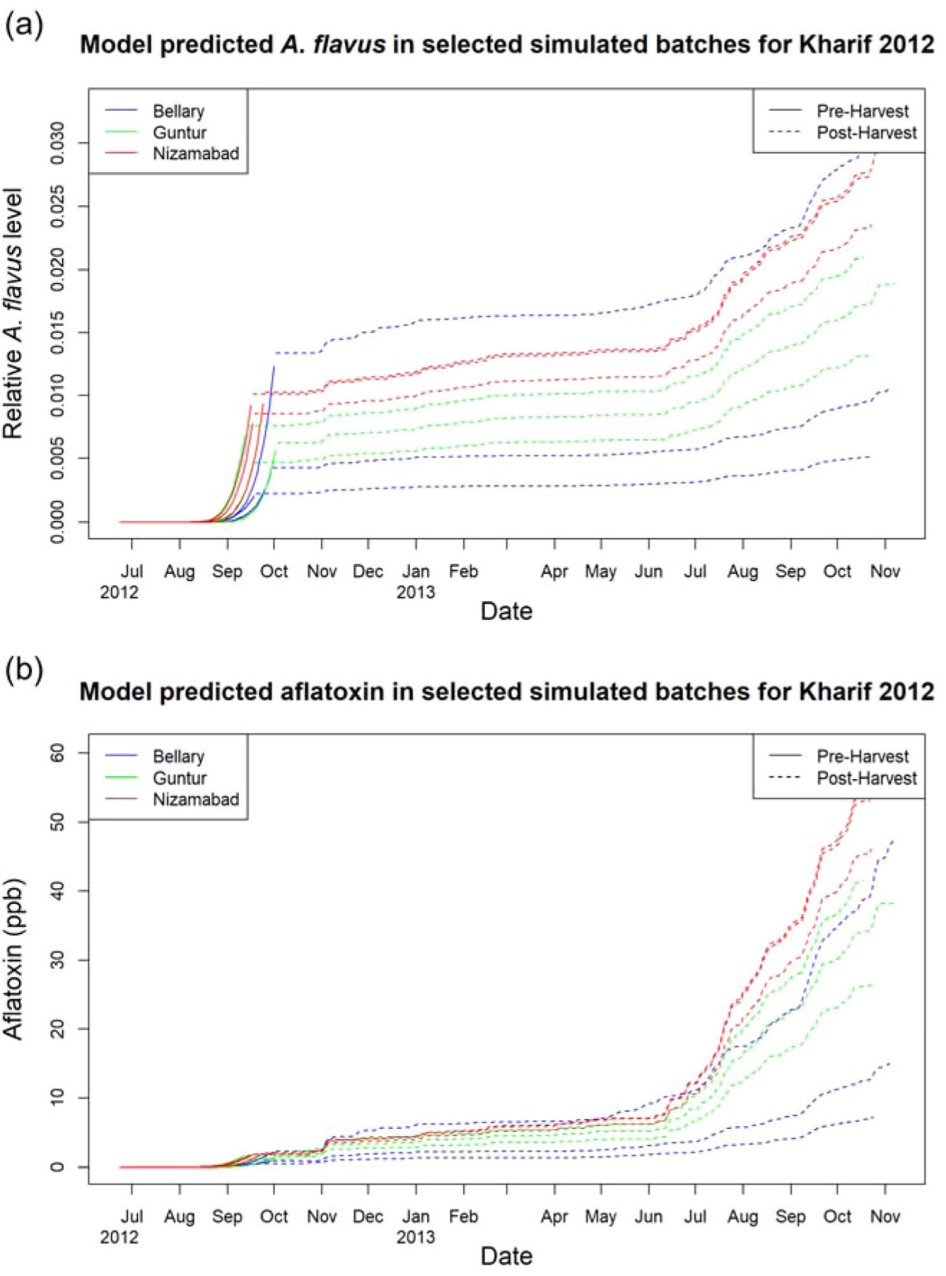
Per shipment time courses. Hourly time course for 11,700, hours of (**a**) *A. flavus* colonisation and (**b**) cumulative aflatoxin production for three selected batches of maize in each of the three sourcing regions, Bellary, Guntur and Nizamabad for the 2012 Kharif growing season with storage extending into 2013.

The model can be used to gain an understanding of the behaviour of the overall system, with insight into predicted levels of *A. flavus* and aflatoxin in batches over time from planting through to storage (Fig. 6). For clarity, we focus on a single cropping season, the 2012 Kharif harvest, and show three individual batches from each of the three sourcing regions; however, the same analysis could be performed on any set of batches from any time periods and locations.

The model outputs indicate a consistent trend amongst sourcing regions with *A. flavus* colonisation increasing rapidly during the maize growing season (June–September 2012) up till harvest, after which substantial *A. flavus* growth occurs during storage (Fig. 6a). Aflatoxin levels increase over time, with most aflatoxin production occurring in the storage phase and very significant increases when left in storage for a long time (Fig. 6b). In the Kharif cropping season using the historic weather data for 2012, the Nizamabad region consistently shows the highest *A. flavus* levels both pre- and post-harvest, followed by Guntur and then Bellary. We note while the general trend is for batches from the Bellary region to have lower *A. flavus* and aflatoxin levels than batches from other regions, one batch in Bellary establishes itself during pre-harvest as significantly more contaminated with *A. flavus*. During storage the *A. flavus* levels in this batch continue to grow and remain the highest out of all in the tracked batches. Aflatoxin levels within this batch are initially the highest out of all tracked batches, however, despite the high *A. flavus* levels, aflatoxin concentration after a year in storage is lower than four of the nine tracked batches as conditions at the market storage location are not as conducive as at other markets.

The different environmental conditions within and between regions can lead to significant differences and divergences in *A. flavus* and aflatoxin levels over time. While batches are dispersed over the region at different farms during the pre-harvest maize growing phase, batches within a region can be subject to different environmental conditions, allowing for a range of different batch statuses by the time of entering storage. Once in storage, batches stored in the same market location will be subject to the same environmental conditions, and hence follow similar trends thereafter. Higher *A. flavus* levels at time of entering storage led to higher *A. flavus* growth rates within storage, and these *A. flavus* levels are the drivers of aflatoxin production during conducive conditions in storage. It is important to note that while the pre-harvest phase does not directly produce as much aflatoxin as long-term storage, it is the final pre-harvest state of a batch determines the initial state in storage, and thus how much aflatoxin will be produced if suitable conditions occur. In addition, the pre-harvest condition is important for accurate predictions of aflatoxin levels in batches delivered after a short period of storage. In addition to delivering insight into the life history of individual batches, the model can also be used to look at regional trends in distributions of *A. flavus* and aflatoxin over many years (Supplementary Fig. 8).

### Application of the model - Nowcasting for decision support

**Fig. 7:**
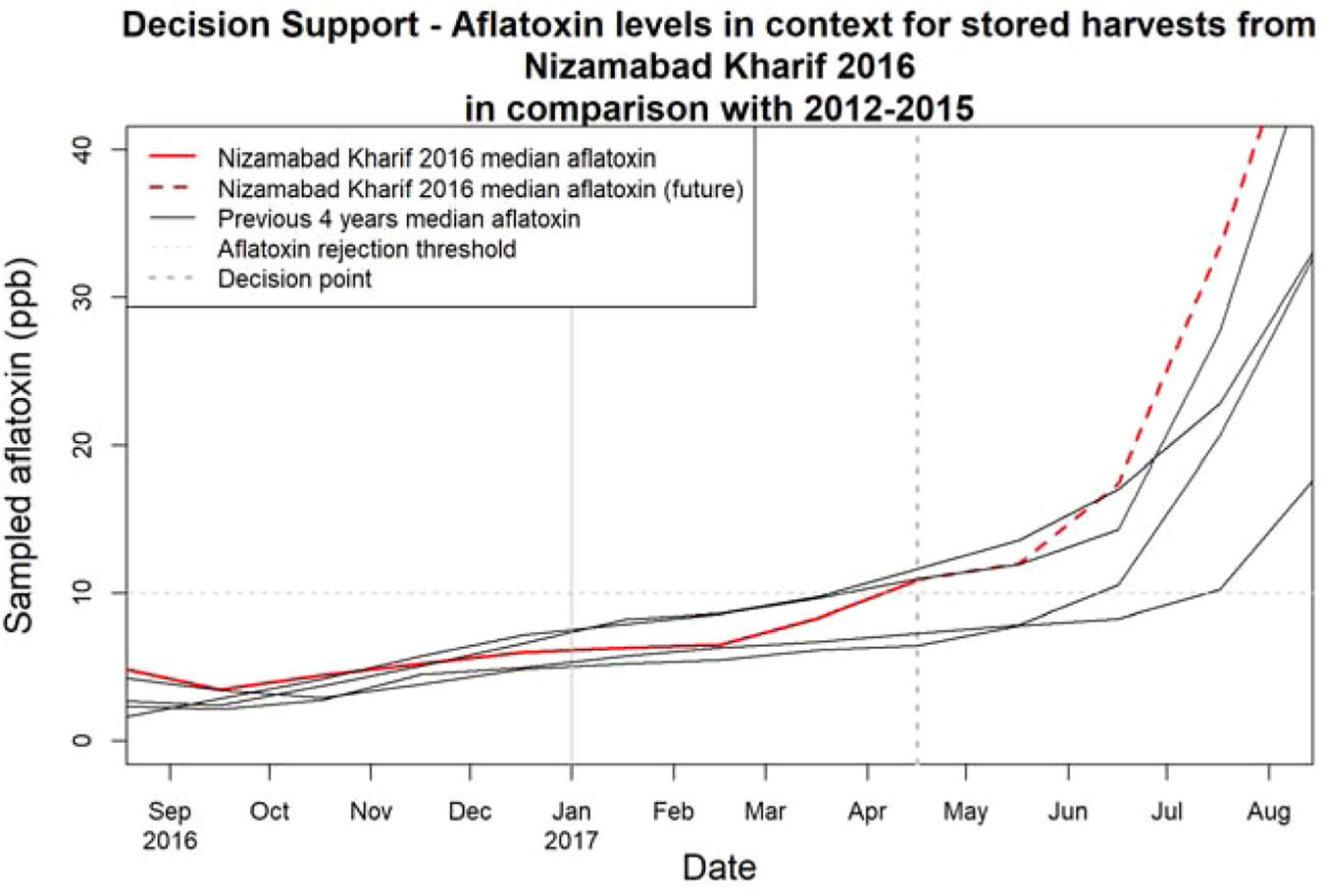
Decision support. Predicted median levels of aflatoxin in simulated batches from the hourly model stored for the Nizamabad Kharif 2016 crop (red) from harvest in August 2016 to August 2017 in context against Nizamabad Kharif batches from the previous four years (black). The dotted vertical line indicates the hypothetical user decision point, with the dashed red line thereafter indicating the at that point unknown future aflatoxin levels. The horizontal dashed line indicates the 10-ppb rejection threshold.

Model predictions can be used for nowcasting to help inform decisions on sourcing using a combination of currently available and historic data for sourcing regions (Fig. 7). For this scenario, we consider a sourcing manager at a major consumer of maize as a hypothetical user of the model in April 2017 (dotted vertical line). The user has observed an increase in rejection rates from the Nizamabad Kharif 2016 shipments during April 2017 (red line). The model indicates that at this point median aflatoxin levels in these stored batches are reaching the 10-ppb rejection threshold. With the model, the user can see likely trends using the predictions for previous years as context. The user would observe that this season is now trending with the worst of the previous four years. Using the previous four years (black lines) as a guide, aflatoxin levels remain similar for another month, but within two months would be predicted to have increased substantially. Based upon this information, the user may decide to source from another crop within at most two months, in order to avoid significantly increasing rejection rates. The dashed red line, which shows the model predictions for the progression of aflatoxin levels after the decision point (Fig. 7), is given for reference. It would not be available to a user at the time of making the decision beyond the availability of future forecast weather data; however, the recent historical ensemble can always be used as a stand in for future trends and variability at a given location. This information would provide value in diverse ways to users at different points in the supply chain: for example, a policymaker could decide when it is necessary to encourage food imports or release food security stockpiles to avoid malnutrition or food price shocks.

### Application of the model: scenario analysis

**Fig. 8:**
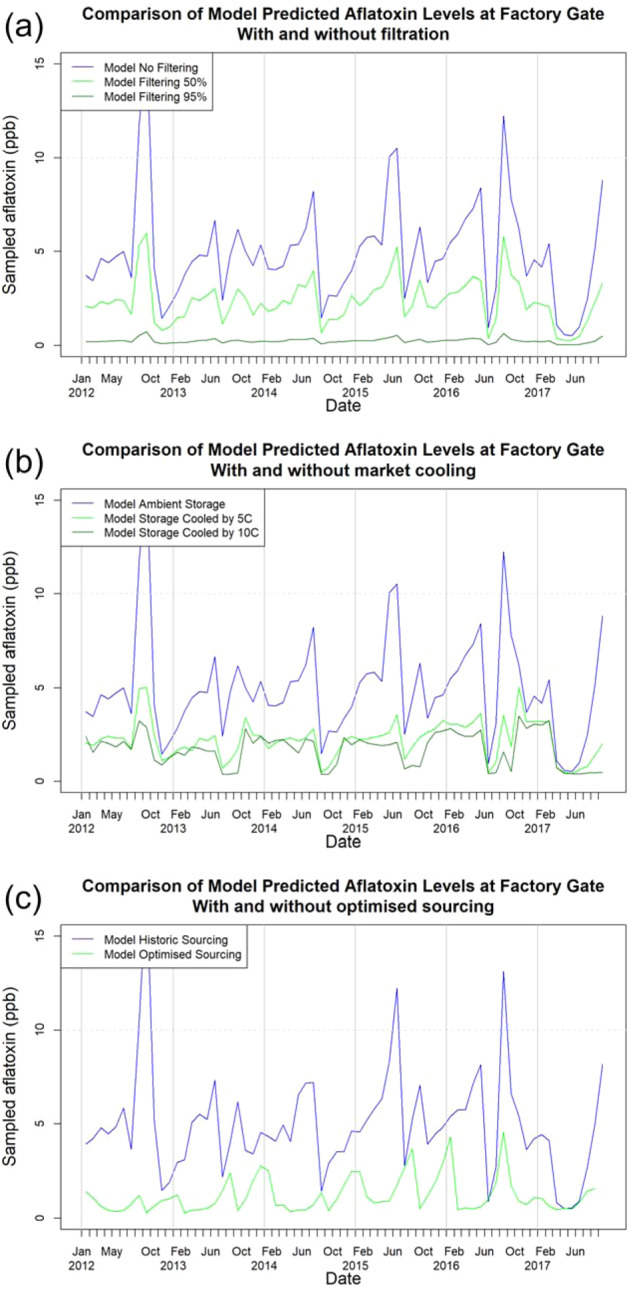
Scenario analyses. Scenario analysis—Using the model to predict the effectiveness of interventions. **a** The baseline model predictions for median aflatoxin levels at the factory gate with no intervention (blue) are compared with the predicted level of aflatoxin that would be observed with the universal adoption of filtering with 50% effectiveness (green) and 95% effectiveness (dark green). **b** The baseline model predictions for median aflatoxin levels at the factory gate with no intervention (blue) are compared with the predicted level of aflatoxin that would be observed with the universal adoption of market storage facilities cooled by 5 ^o^C below ambient temperatures (green) and 10 ^o^C below ambient temperatures (dark green). **c** The observed aflatoxin levels at the factory gate using historic sourcing (blue) are compared with the model predicted aflatoxin levels from using the model predicted optimal sourcing strategy (green). The deliveries from the hourly resolution model are shown aggregated to months for plotting purposes.

The model can be used to simulate and analyse the effects of potential interventions to reduce the risks of contamination. We examine the effects of filtration at harvest time (Fig. 8a), controlled storage (Fig. 8b) and alternative regional sourcing arrangements (Fig. 8c) on the time profile of aflatoxin risk observed at the factory gate. For the scenarios in Fig. 8a, b), we assume that the interventions are applied uniformly within all markets and sourcing occurs as historically. For the alternative sourcing regions scenario (Fig. 8c) there are no interventions beyond the changes to regional sourcing profiles. For consistency, the analyses compare interventions with a baseline involving no intervention. We note that the model can be used to examine any combination of interventions, however for brevity we restrict the analyses here to single factors.

The improved post-harvest filtration scenarios show substantial reductions in aflatoxin levels at the factory gate, with increasing impact as the filtration effectiveness increases (Fig. 8a). Filtration to remove contaminants is most effective when aflatoxin levels are highest, with proportionately lesser effects for lower aflatoxin levels. Due to the filtration decreasing both *A. flavus* and aflatoxin levels proportional to filtration effectiveness, the overall effects on the time profile of factory gate aflatoxin levels are roughly linear (Fig. 8a). Without additional experimental data, it is not known how practically feasible it is to achieve the levels of filtration corresponding to the parameters chosen here.

Controlled storage scenarios also indicate significant benefits in reducing the levels of aflatoxin at the factory gate, with increasing effect as temperature is decreased below ambient conditions (Fig. 8b). The cooled storage examined here simulates a cooling system with limited effectiveness, which is able to reduce temperatures by a fixed amount below ambient temperatures, but not capable of maintaining a fixed temperature. In contrast to filtration, cooling of storage has a non-linear effect, with 5 C temperature reductions having a proportionately greater effect than 10 C, albeit 10 C dampens fluctuations in the median response compared with the ambient and 5 C scenarios. However, in some cases, such as mid-2016, 10 C cooling provides no further benefit as the cooled storage cannot reduce aflatoxin levels below those at which the batch begins storage. It should be noted that it is theoretically possible, although unlikely, for limited cooling of storage to make conditions more suitable for *A. flavus* growth and aflatoxin production in conditions when the ambient temperature is above 40 C. In reality, this could be avoided by deactivating the cooling systems in these situations, and this was not thought to be a common enough occurrence to incorporate into the model.

The original purpose of the model is to understand and predict aflatoxin levels at the factory gate when sourcing from a variety of sourcing areas, with material from these areas demonstrating higher and lower aflatoxin risks over time. Therefore, a natural question for industry to ask is whether an alternative sourcing strategy might be able to obtain material lower in aflatoxin by sourcing from different areas to those used historically. To examine the potential effects of modified and optimised sourcing strategies the model was used to generate a sourcing strategy constructed to have been optimal for the historical period. This optimal sourcing strategy was generated by first running a realisation of the hourly model to analyse all potentially available material at all markets each month, taking account of weather data for each sourcing region up to that time. An optimal sourcing strategy is then constructed to source each month from the available batches with the lowest predicted aflatoxin. The model is then run again using this optimised sourcing strategy to generate the predicted aflatoxin levels under this strategy (Fig. 8c). The optimal sourcing strategy typically greatly reduces the observed levels of aflatoxin compared with the historical sourcing profile (Fig. 8c). The annual profile of aflatoxin levels, especially peak levels is significantly changed, with most of the highest peaks in aflatoxin levels being removed under the optimal sourcing strategy. In only four of the 70 months analysed did historic sourcing match the performance of the optimal sourcing strategy. The optimal sourcing aflatoxin profile acts as a theoretical lower bound for the effectiveness of a sourcing strategy, as any alternative sourcing strategy could achieve aflatoxin levels equivalent or worse than those observed here. In practice, a user in industry could use the model to identify areas at lower risk for aflatoxin historically as well as estimate the status of currently stored material in order to identify regions of lower risk when making sourcing decisions.

## Discussion

The implications of aflatoxin contamination in the supply chain are far reaching for human and animal health^32^ with additional concerns about enhanced risks associated with climate change^16,33^. Hence, a considerable amount of effort has been expended in linking experimental with modelling work to predict risk^12,14,18,34–37^. Increasing attention has also been focused on the potential for aflatoxin predictive risk modelling for low and middle income countries, especially in sub-Saharan Africa^18^. Despite raised awareness and tools for risk prediction, unexpected severe aflatoxin contamination events still occur^38,39^. Most risk prediction models have focused on elucidating weather and site variables on the pre-harvest dynamics of *A. flavus* growth and aflatoxin production^18^. Approaches range from empirical methods involving statistical fits of linear models^40^ through empirical models coupled with crop growth dynamics^36^ to more mechanistic dynamic models exemplified by the AFLA model originally developed by Battilani^12^ that has been widely used^12,14–16,35^ including adaptation for pistachio crops^41^. The integrated mechanistic model that we present here provides coverage of the entire supply chain from planting to delivery, so allowing for continued fungal growth and toxin accumulation after harvest.

Building on the work of Battilani and co-workers^12,14,35^, the integrated mechanistic model provides a theoretical framework that tracks the dynamics of *A. flavus* growth and aflatoxin production throughout the supply chain (Fig. 4) across a heterogeneous sourcing region (Figs. 2, 5). The framework facilitates analysis of an optimised sourcing strategy (Fig. 8c). It also allows scenario analysis, for example to compare the effectiveness of different intervention strategies to minimise the risks of aflatoxin contamination at the end of the supply chain. By integrating explicit biological and intervention processes into the modelling framework (c.f. Fig. 3) a mechanistic model allows sensitivity and scenario analyses. This is done by adjusting tuneable parameters (Table 1) that map onto recognisable processes via the model equations (Supplementary Table 1): for example, in modelling the effects of changing temperature on aflatoxin production rates.

While the model was originally intended to give insights and inform decision-making for those responsible for large-scale sourcing of maize, there are other potential users. The model predictions can be used in the agricultural finance sector to reduce risk to creditors and by large-scale consumers and distributors to target sourcing and purchasing of maize for direct consumption and for processing. More efficient pre-allocation of risk weightings to crops would help guide the agricultural sector to plan the allocation of resources for testing and evaluation of maize consignments in the short term and to plan for the introduction of storage technologies to reduce contamination improve health outcomes and increase profitability. Care needs to be given to reduce the risks of unintended consequences in which smallholders may be selected against according to model predictions. We envisage that this can be offset by using the model to provide guidance in managing the agronomy and on-farm storage of the crop to reduce risks of aflatoxin production.

The absence of data for fitting at intermediate stages in the supply chain, other than information about sourcing regions in shipments arriving at the processing factory, rendered model fitting and parameterisation challenging. Nevertheless, it was possible to estimate five epidemiologically important parameters (Table 1) using ABC from the time-series for aflatoxin levels of shipments sampled at the factory gate. The available data admit a range of equally plausible parameters, which have a small degree of difference in terms of predicted model outcomes. Accordingly, there were inevitable trade-offs amongst posterior distributions for certain parameters in the fitting (Supplementary Fig. 1). Nevertheless, the time series for aflatoxin levels from the parameterised model successfully captured the overall profile, scale and variance of the historical aflatoxin datasets used for fitting (2012–15) and validation (2016–17) (Fig. 4a–c), meeting the original objective of predicting observed aflatoxin levels at the factory gate. With more data, particularly at points earlier in the supply chain, it would be possible to refine the parameter estimation and reduce uncertainty in model outcomes. Data for *A. flavus* levels at any points in the supply chain would provide significant value in validating and refining the model. The spatial and temporal variability in suitability for *A. flavus* and aflatoxin production highlighted by the risk maps produced by our model (Fig. 5) highlight the importance of considering the location history and timing of movements of batches of maize when assessing aflatoxin risk.

The integrated model prediction accuracy of 85% for median aflatoxin levels within $\pm$ 4ppb of historical observations consistently across fitting and validation datasets shows the potential utility of the model. The integrated model was constructed and validated to predict aflatoxin values at the factory gates rather than rejection rates about a specific threshold value. The ability of the model to capture the trends in rejection rates at the processing factory due to aflatoxin levels above the required limit of 10-ppb was therefore an additional test of the potential utility of the model. Accurate predictions of rejection rates and the likelihood of aflatoxin levels exceeding rejection thresholds are also of particular interest to the intended users of the model outputs. We set a comparatively strict criterion, whereby monthly rejection rates for the model were classified as accurate only if they were within $\pm 10 \%$ of the observed monthly rejection rates. This criterion gave an accuracy of 50% with the model being significantly more likely to overpredict than underpredict rejection rates. As a consequence, the integrated model is conservative: having a substantially lower frequency of false negatives, which are potentially dangerous when undetected, than false positives. Overestimation of rejection rates tended to occur during periods when the historical periods were low. Overestimation may tend to unfairly mark certain regions as higher risk, with negative effects on marketability from these areas, but this effect could be mitigated with increased ground truthing in target areas, with these test results used to refine the model. Various levels of accuracy have been quoted for predictive models of *A. flavus* aflatoxin production (see e.g. the recent review by Keller et al.^18^) but each of these has to be analysed in relation to the strictness of the criterion, the variable under consideration and the detailed methods used to assess the accuracy. A detailed analysis amongst the models would be beneficial but is beyond the scope of the current paper. One practical way of assessing the utility of the integrated and other models is in the use of the models in decision making. Especially when viewed from the perspective of high-level decision making about sourcing regions, the model offers a valuable tool to decision makers in industry. The integrated model is currently being tested by Mars Inc. at multiple worldwide sites with the intention to report in due course.

In developing an integrated model, we incorporated many of the assumptions of the AFLA model^12–14,35^ in both the pre-harvest and the post-harvest components. Details of the functions underlying the model together with the variables and parameters are summarised in detail in Supplementary Table 1. The assumptions underpinning the integrated model introduced in this paper and other models^18^ merit further detailed experimental and theoretical investigation. For example, soil moisture conditions, regarded by Chauhan et al.^36,40^ as important during the pre-harvest phase are not included in the integrated nor the AFLA model^14^. In turn, in applying a model for pre-harvest dynamics of *A. flavus* and aflatoxin production in Kenya, Chauhan et al.^36^ excluded sporulation and spore germination under an assumption that *A. flavus* fungal inoculum was always available for infection. Further work is needed to analyse the importance of these various assumptions on aflatoxin risk under different field conditions. Flexible modelling frameworks complemented by appropriate time course data provide a means to do this.

The choice of meteorological data at a 10 km resolution was driven by availability of data, with this being the best readily available data source covering the target spatial extent and time frame available when conducting the study. The model is capable of using input data at any resolution that a user can source, so going forward a user could use their preferred data source at the best resolution that is available or incorporate data from multiple sources as needed for their target locations. Given the available testing data only provided kernel moisture measurements at the point of delivery, a decision was made not to explicitly model kernel moisture levels in the integrated model. While aflatoxin levels would strictly accumulate in a kernel over the lifetime of a shipment, kernel moisture levels can be significantly increased or decreased in short time windows, and so the final moisture measurement does not provide a reliable measure of the conditions experienced by the shipment over its lifetime. Currently, our model uses plant growth stage to determine moisture content pre-harvest and uses an assumption of an equilibrium between environmental humidity and surface moisture levels in the kernels while in storage, essentially a model of ambient surface conditions. More measurements of moisture levels in shipments throughout the supply chain could be used to validate an explicit kernel moisture model component. With explicit kernel moisture modelling, the post-harvest drying intervention could be modified to affect kernel moisture levels explicitly, in place of the current drying protection time proxy. In addition, weather conditions at the time of harvest could inform the effectiveness of outdoor drying, as well as potentially be used to predict severe *A. flavus* blooms from material subjected to heavy rainfall at harvest. We note that farmers have free choice over the precise timing of harvest for a mature crop and the method of drying coupled with the very short time frames of precipitation events. Accordingly, we used relative humidity as a driving variable rather than rainfall in the pre-harvest model. The model could be adapted to include the rainfall at harvest times by allowing for an additional high-moisture period following wet harvests, albeit at the cost of incorporating an extra function for drying with a rate parameter that would require estimation. Although beyond the scope of the current work, we recommend this as an area for further study. We note that as precise harvest timing is within control of the farmers, this is a potential area for providing direct model decision support to farmers looking to minimise contamination of crops.

An important objective in modelling the entire supply chain from field through storage to factory gate was to incorporate intervention strategies to enable scenario testing. Our results show the likely impacts of filtration and cooling during storage as well as risk-based optimisation of sourcing regions to reduce the overall risk of aflatoxin contamination at the factory gate (Fig. 8). Opportunities to test the utility of the integrated model to assess aflatoxin risk on maize, to inform sourcing decisions and to compare intervention strategies are currently underway by Mars Inc at a range of worldwide sites with different growing and storage conditions. The components of the model are designed to be readily adaptable to different locations in the world by including components to explicitly incorporate changing environmental conditions or cultural practices such as bags, storage conditions or other processing methods.

In summary, we have developed and tested an integrated model for aflatoxin contamination in maize supply chains from planting to harvest and storage through to delivery. The model is valuable in assessing and predicting risks of aflatoxin contamination and allowing users to make decisions based on likely aflatoxin levels. The model also allows exploration of management options that reduce risk. The utilisation of the model in industry by Mars Inc. commenced in 2020 and continues to provide valuable insight. Important areas for future work are work are in gathering data for aflatoxin, *A. flavus* and kernel moisture levels at more points during the supply chain to allow additional refinement and validation of the model and understanding the dynamics of kernel moisture during storage. Almost all further extensions of the current work would inevitably require additional data to test and validate the usefulness of models for decision making. The general model predictions would benefit from aflatoxin measurements at a point in the supply chain earlier than delivery, particularly at the beginning of storage. The model uncertainty around *A. flavus* levels could be reduced by *A. flavus* measurements at two points in the supply chain, preferably at the start of storage at the market in addition to the current measurement taken at the time of delivery. Moisture measurements at multiple points in the supply chain, especially prior to delivery, would be beneficial. It is not immediately obvious if adding an explicit kernel moisture model component would improve model performance. Turning to model scenarios analyses, these can be combined with an economic analysis in order to select those interventions with the greatest economic return on investment. To do so would require further information on the costs of providing interventions, such as filtration or cooling equipment. Finally, it is possible that it is not economically optimal to provide the lowest achievable aflatoxin levels, and a user may prefer to explore scenarios in order to reliably achieve acceptably low aflatoxin levels below the 10-ppb threshold for a lower cost.

### Reporting summary

Further information on research design is available in the Nature Research Reporting Summary linked to this article.

### Supplementary information

Reporting summary

Supplementary Information: An integrated model for pre- and post-harvest aflatoxin contamination in maize

## Data Availability

The datasets associated with this study have been deposited at 10.5281/zenodo.8026592. The authors declare that all data supporting the findings of this study are available in the paper, Supplementary Information and the above repository.

## Code availability

The code associated with this study has been deposited at 10.5281/zenodo.8026592. The authors declare that all code supporting the findings of this study is available in the paper, Supplementary Information and the above repository.
