# Supplementary material for: An integrated model for pre- and post-harvest aflatoxin contamination in maize
Source: NPJ Sci Food. 2023 Nov 18;7:60. doi: 10.1038/s41538-023-00238-7 (PMC10657429; doi:10.1038/s41538-023-00238-7)
Supplement: Supplementary file 2 — Supplementary Information: An integrated model for pre- and post-harvest aflatoxin contamination in maize [file 41538_2023_238_MOESM2_ESM.pdf]

Supplementary Information:
An integrated model for pre- and post-harvest aflatoxin contamination in maize

Richard O.J.H. Stutt[1*], Matthew D. Castle[1,2], Peter Markwell[3], Robert Baker[3], Christopher A. Gilligan[1]

[1]Epidemiology and Modelling Group, Department of Plant Sciences, University of Cambridge, Downing Street, Cambridge, CB2 3EA, UK.

[2]Cambridge Centre for Data-Driven Discovery, Department of Genetics, University of Cambridge, Downing Street, Cambridge, CB2 3EH, UK.

[3]Mars Global Food Safety Center, Mars Inc., Yanqi Economic Development Zone, Huairou, Beijing, China.

*Email: rs481@cam.ac.uk

Supplementary Methods

Summary of model structure

Additional details of the structure of the integrated models for pre- and post-harvest model on *A. flavus* dynamics and aflatoxin accumulation are summarised below. The principal, variables, parameters and function used in the full model incorporating pre-harvest, intervention and post-harvest structure are summarised in Supplementary Table 1.

Pre-harvest model

Maize Growth Model

We follow Shaykewich [1] and Yan and Hunt [2] in modelling maize growth using an accumulated thermal unit (growing degree day: GGD). The GDD [3] are calculated from the meteorological data for the different sourcing regions in Karnataka, Pradesh and Telangana. The model accounts for changing susceptibility of maize to *A. flavus,* from the appearance of silks (GDD ≥ 780 ^{0}day) increasing to a maximum and decreasing as the crop matures. The model also allows for the effects of heat stress on maize development (since the maize relative growth rate has an optimum value at 30°C and drops to zero at and above 41°C).

Following Battilani et al. [4], the model distinguishes a sequence of biological processes for the growth of *A. flavus* and aflatoxin production on the maize crop, each of which is driven by different environmental conditions calculated from the spatially-resolved meteorological data for the location of fields within the sourcing regions (Supplementary Table 1).

Sporulation

We assume that soil-borne populations of *A. flavus* are endemic within the maize growing regions of Karnataka, Andhra Pradesh and Telangana with an environmentally conditioned sporulation rate, α, given by the following equation (after Battilani *et al.*, 2013):

$$\alpha = \alpha_0\left(5.28\left(\frac{T-5}{40}\right)^{2.05}\left(1-\frac{T-5}{40}\right)\right)^{0.98} \times aw_s{}^{18.59} \quad , \tag{1}$$

where T is ambient temperature (C^0) and aw_s is ambient water activity, which is based upon relative humidity, rainfall and temperature (see Supplementary Table 1 for the underlying calculations); α_0 is a scaling parameter that converts the relative sporulation rate given in Battilani *et al.* [4] to an absolute sporulation rate and is estimated directly from the time series data.

Spore liberation and deposition

Spore liberation and deposition is a complex process that requires information on relative humidity and local, small-scale wind speeds Li & Kendrick [5]. The rate of spore liberation is modelled using environmental thresholds for leaf

wetness/dew formation. Following Battilani *et al.* (2013), we assume that fungal conidiophores can only liberate spores when not covered by a layer of water or dew and so we adopt the extended threshold model for surface moisture proposed by Kruit et al. [6], with the assumption that all available spores are liberated when there is no surface moisture and no spores are liberated when surface moisture is present. See Kruit *et al.*, (2004) and Supplementary Table 1 for the exact description of the liberation rate equation. Sporulation rate is set to π = 0 prior to silk emergence (the point at which silks are assumed to have emerged). Thereafter we assume that the proportion of spores that are deposited on the plants is constant, π_0. Without loss of generality this parameter is set to 1.

Spore Germination

Spore Germination was treated a binary process by Battilani *et al.* (2013) who identified a threshold in ambient water activity – temperature space above which germination was observed to occur. We use this threshold to determine whether all successfully deposited spores are permitted to germinate or not in a given time period. (Supplementary Table 1)

Infection and susceptibility

After silks emerge maize susceptibility varies with development stage [7]. Data compiled by Sydenham et al. [8] and Siriacha et al., (1989) were used to construct an equation for relative maize susceptibility to *A. flavus* infections, σ, (Figure 1 main text ; Supplementary Table 1)..

Aspergillus flavus growth

Following Battilani et al., (2013), the growth rate of *A. flavus*, β, is given by.

$$\beta^{pre} = \beta_0^{pre}\left(5.98\left(\frac{T-5}{43}\right)^{1.7}\left(1-\frac{T-5}{43}\right)\right)^{1.43} \times \left(\frac{1.12}{1+e^{(27.37-30.08\,aw_i)}}\right), \qquad (2)$$

where *T* is the ambient temperature in Celsius and aw_i is the within kernel water activity level, which in turn is governed by the growth stage of the plant with water activity falling as the maize crop matures (see Supplementary Table 1 for details). The scaling parameter β_0^{pre} converts the relative growth rate given in Battilani et al. [4] to an absolute growth rate and is estimated directly from time series data at the factory gate.

Aflatoxin production

Again following Battilani et al., (2013), the aflatoxin production rate, τ, is given by,

$$\tau = \tau_0\left(4.84\left(\frac{T-10}{37}\right)^{1.32}\left(1-\frac{T-10}{37}\right)\right)^{5.59} \times e^{(30.08\,aw_i-32.8)} \quad , \qquad (3)$$

where *T* is ambient temperature in Celsius and aw_i is the within kernel water activity level; τ_0 is a scaling parameter that converts the relative aflatoxin production rate given in Battilani et al., (2013) to an absolute production rate and is estimated directly from data.

Intervention Model

See main text for details and Supplementary Table 1.

Postharvest model

Colonisation and growth

Growth of *A. flavus* on the maize kernels occurs from *A. flavus* present on the kernels (*F*) on other contaminant material (fines) within the bag (*X*). We assume a common rate constant β_0^{post} that is modulated by environmental conditions with a functional form adopted from Battilani *et al.* [4] .

$$\beta^{post} = \beta_0^{post}\left(5.98\left(\frac{T-5}{43}\right)^{1.7}\left(1-\frac{T-5}{43}\right)\right)^{1.43} \times \left(\frac{1.12}{1+e^{(27.37-30.08\,aw_s)}}\right), \qquad (4)$$

where β^{post} follows the same form as the pre-harvest model; however, here the water activity, aw_s, is determined by ambient humidity levels, rather than the internal moisture content of the plant. The water activity, aw_s, is taken to be the maximum of the water activity due to humidity and dew point. Supplementary Table 1 for details. The parameter β_0^{post} is obtained by fitting the integrated model to time series data.

Aflatoxin production

The post-harvest aflatoxin production rate (τ) follows the form as for the pre-harvest model (c.f. Supplementary equation.2, with aw_s now related to ambient humidity levels. The rate constant τ_0 is common between the pre- and post-harvest models, as the fitting process determined separate rate constants provided no significant benefit (see section Model parameter estimation).

Model Parameter estimation

The ABC fitting was performed by taking 750,000 independent samples from the prior and calculating the fitting metric € for each simulation. The ABC rejection tolerance was chosen to accept the top one percent of simulations according to the metric and reject all others. For all parameters except the aflatoxin production rate (τ) the prior was uniform over independent intervals with bounds as shown in Supplementary Figure 1. These bounds were chosen based on preliminary simulation runs. The choice was made not to explore values for the post-harvest aflatoxin growth rate (β^{post}) below $\log(\beta^{post}) = -4$ as below this value there is effectively no post-harvest aflatoxin growth, and this was deemed biologically implausible. Due to the linear effect of τ, this parameter was chosen by first sampling all other parameters from the prior and running a simulation with these parameters and a value of τ = 1. The actual value of tau was then obtained via choosing via interval bisection a rescaling for the simulated aflatoxin values such that the fitting metric (E) was minimised. Further exploration of the tau dimension was achieved by taking a sample of 20 values of tau around the obtained optimum ranging from half to double the optimal value. The choice to sample τ in this way was made in order to reduce the effective dimensionality of the parameter space being searched, greatly reducing computation resources needed to achieve a given useful sampling density.

The fitting process has established bounds for the model parameters. Some parameters are correlated, and with more data it may be possible to disentangle and further constrain the parameters. The data required to achieve this would require monitoring at other points in the supply chain prior to final delivery and information on *A. flavus* levels.

While model *A. flavus* levels are within reasonable bounds, the effect of the primary sporulation rate (α) and aflatoxin production rate (τ) is relatively linear, allowing these parameters to trade off against each other freely, setting the scale of *A. flavus* values. The pre-harvest aflatoxin growth rate (β^{pre}) also has a relatively simple ratio effect while *A. flavus* levels remain in the exponential growth phase, explaining the trade-off with α. Having information about *A. flavus* levels at multiple points in the life cycle of a batch, optimally at the start and end of storage, would constrain *A. flavus* levels and allow these trade-offs to be eliminated, much more tightly constraining these parameters. While these additional data would significantly constrain parameters and model *A. flavus* predictions, it would not constrain or change model aflatoxin outputs significantly, as the aflatoxin levels are again linearly scaled by τ and thus any values of α and (to a reasonable degree) β^{pre}can be compensated for by τ.

Fitting independent aflatoxin production rate parameters for the pre- (τ_0^{pre}) and post-harvest (τ_0^{post})model components was attempted, but model performance was unchanged and the aflatoxin production rate parameter values were highly correlated along the line $\tau_0^{pre} = \tau_0^{post}$. Hence, without a biological motivation to separate these rate parameters, a decision was made to use a common aflatoxin production rate parameter in order to reduce the dimensionality of the parameter space and reduce computational requirements.

Supplementary Tables

Supplementary Table 1 Summary of model structure. Summary of the principal variables, parameters and functions used in the integrated model for the dynamics of *A. flavus* growth and aflatoxin production during the supply chain for maize incorporating pre-harvest, intervention (postharvest) and post-harvest stages.

Parameters/variables	Symbol	Function / explanation	Reference
		Pre-harvest fungal and aflatoxin dynamics	
Sporulation rate	α	$\alpha = \alpha_0 \left(5.28\left(T_{eq}(T,5,45)\right)^{2.05}\left(1-T_{eq}(T,5,45)\right)\right)^{0.98} \times aw_s{}^{18.59}$	Battilani et al. [4]
Spore liberation rate	λ	$\lambda = \lambda_0 * dew(RH, \Delta RH)$	Kruit *et al.* [6]
Spore deposition proportion	π	π_0 * σ	
Spore germination rate	γ	$\gamma = \begin{cases} 1 & \text{if } aw_s > 0.0004\,T^2 - 0.0261\,T + 1.2469 \\ 0 & otherwise \end{cases}$	Battilani *et al.* [4]
Host susceptibility (silk availability)	σ	$\sigma = \begin{cases} \left(\frac{1}{4.1 \times 10^{21}}\right)(GDD_c - 670)^{4.0}(1700 - GDD_c)^{3.9} & \text{for } 670 < GDD_c < 1700, \\ 0 & otherwise \end{cases}$	Shaykewich [1]<br>Siriacha *et al.* [7]<br>Sydenham et al. [8]<br>Battilani *et al.*[9]
Fungal growth rate (pre-harvest)	β^{pre}	$\beta^{pre} = \beta_0^{pre}\, G(T, W = aw_i)$	Battilani *et al.* [4]
Aflatoxin production rate (pre-harvest)	τ	$\tau = \tau_0\, K(T, W = aw_i)$	Battilani *et al.* [4]
		Post-harvest management interventions	
Contaminant rate	μ	μ = 0	
Filtering efficacy for removal of contaminants	ψ	ψ = 0	

Drying protection period	δ	δ (Estimated)	
Colonisation rate	η	$\eta_0 = \beta_0^{post}$	
		Post-harvest fungal and aflatoxin dynamics	
Fungal Growth rate (post-harvest)	β^{post}	$\beta^{post} = \beta_0^{post} G(T, W = aw_s)$	Battilani *et al.* [4]
Aflatoxin production rate (post-harvest)	τ	$\tau = \tau_0\, K(T, W = aw_s)$	Battilani *et al.* [4]
		**State variables that evolve according to model dynamics (pre/post-harvest)**	
Growing degree days	GDD	Accumulated Growing degree days (pre-harvest only)<br>GDD (Growing Degree Days) = hourly accumulated integral of the instantaneous temperature dependent growth rate, θ<br>$\theta = \begin{cases} 1.98425 \times 10^{-6}(41 - T)T^{3.1} & \text{for } 0 < T < 41, \\ 0 & otherwise \end{cases}$	Shaykewich [1]<br>Siriacha *et al.* [7]<br>Battilani *et al.*[9]
Conidia in soil	N_{soil}	Current level of viable conidia available in soil (pre-harvest)	
Conidia on silk	S_{silk}	Current level of viable conidia on silks (pre-harvest)	
A. flavus	$F_{(H, S)}$	*Aspergillus flavus* amount at harvest (H), storage (S)	
Aflatoxin	$A_{(H, S)}$	Aflatoxin level (ppb) at harvest (H), storage (S)	
Contamination	$X_{(H, S, B)}$	Contaminant amount of *A. flavus* at harvest (H), storage (S) and from use of contaminated bags	
		**State variables that are not dynamically evolved**	
Location		Current location of the batch. Used to determine which meteorological data should affect the batch. Piecewise constant with a change when moved from farm to market	
Market		Name of market catchment area the batch resides within. Used to determine which market location to move bag to after harvest.	

Season		Cropping season for which the batch was planted (e.g., Nizamabad Kharif 2015). Used when determining sourcing for factory deliveries.	
Harvest Date		Record of the date on which the batch was harvested	
Drying Protection Date		Date up to which the drying process will be effective at stopping *A. flavus* growth / aflatoxin production.<br>Drying Protection Date = Harvest Date + δ	
Market Date		Date on which the batch is moved from the farm to the market.	
Expiry Date		Date on which (if not yet sold) the crop will be disposed of.	
**Driving variables**		**Meteorological driving variables**	
Temperature (^{0}C)	*T*	Hourly temperature measurements within the ~10km meteorological grid	
Relative humidity (0-100%)	*RH*	Hourly relative humidity measurements within the ~10km meteorological grid	
		**Derived/Intermediate variables**	
Change in RH over 30 minutes (%)	ΔRH	$\Delta RH_t = RH_t - RH_{t-0.5}$	
Availability of moisture from dew formation	$dew(RH, \Delta RH)$	$dew(RH, \Delta RH) = \begin{cases} 1 & \text{if} \quad \mathrm{RH} < 70 \\ 0 & \text{if} \quad \mathrm{RH} > 87 \\ 1 & \text{if} \quad 70 < \mathrm{RH} < 87 \text{ and } \Delta\mathrm{RH} < -2 \\ 0 & \text{if} \quad 70 < \mathrm{RH} < 87 \text{ and } \Delta\mathrm{RH} > 3 \\ \frac{3 - \Delta\mathrm{RH}}{5} & \text{if} \quad 70 < \mathrm{RH} < 87 \text{ and } -2 < \Delta\mathrm{RH} < 3 \end{cases}$	
Ambient moisture content	*MC*	$MC(T, RH) = 2.724199 - 0.0774088\ \mathrm{T} + 0.3480181\ \mathrm{RH} + 0.001073854\ T^2 - 0.003725816\ RH^2 + 0.00002612877\ RH^3 - 0.001080356\ \mathrm{T} * \mathrm{RH}$	
Ambient water activity	*WA*	$WA(MC, T) = \frac{1}{100} \frac{(\log(MC) - 2.09 + 0.011 \times T)}{(0.014 + 0.000049 \times T)}$	

Water activity within the plant	aw_i	$aw_i = 1 - e^{-17.2e^{-0.0012*GDD}}$	Chaylan and Esna-Ashari [10]
Water activity on the surface of kernels due to either ambient water activity or dew	aw_s	$aw_s = \max\,(WA, dew(RH, \Delta RH))$	
Temperature equivalent, a linearised clamped temperature transformation.	T_{eq}	$T_{eq}(T, T_{min,} T_{max}) = \begin{cases} 1 & if\ T > T_{max} \\ 0 & if\ T < T_{min} \\ \dfrac{T - T_{min}}{T_{max} - T_{min}} & otherwise \end{cases}$	
Fungal growth function	$G(T, W)$	$G(T, W) = \left(5.98\left(T_{eq}(T, 5, 48)\right)^{1.70}\left(1 - T_{eq}(T, 5, 48)\right)\right)^{1.43} \times \left(\dfrac{1.12}{1 + e^{(27.37 - 30.08\ \mathrm{W})}}\right)$	
Aflatoxin production function	$K(T, W)$	$K(T, W) = \left(4.84\left(T_{eq}(T, 10, 47)\right)^{1.32}\left(1 - T_{eq}(T, 10, 47)\right)\right)^{5.59} \times e^{(30.08\ \mathrm{W} - 32.8)}$	
Bag Sampling function	B(A)	For a given predicted aflatoxin level, A, the bag sampling result, B, is taken as an exponential random variate with mean A: $$B \sim \exp\,(\lambda = \frac{1}{A})$$	

Supplementary Figures

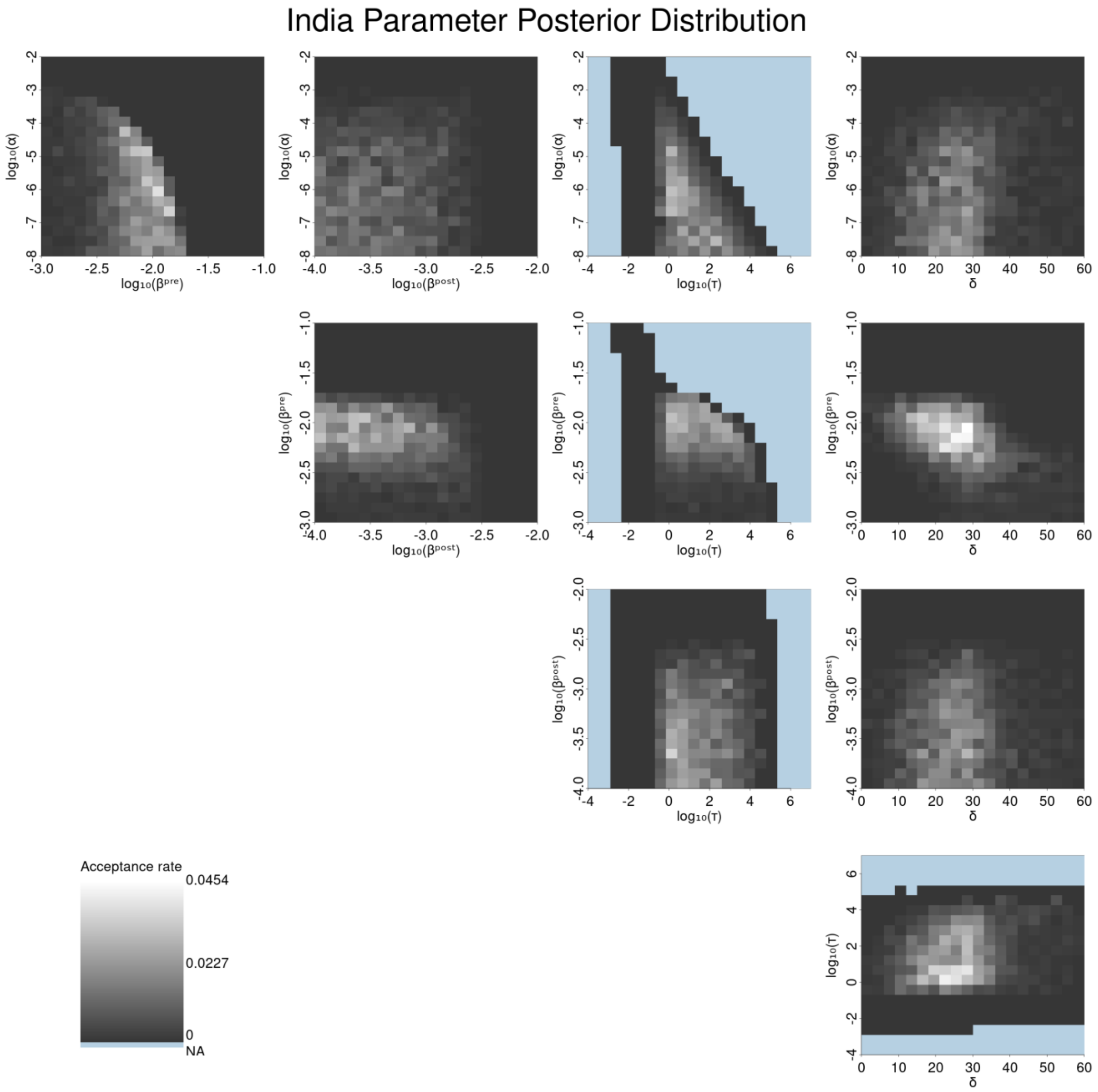

Supplementary Figure 1 Visualisation of the 5-parameter posterior distribution obtained from ABC fitting of the model to historical data from 2012-2015. The subplots show all unique pairwise combinations of two-dimensional projections of the 5-parameter distribution. While parameter values are sampled continuously during the fitting process, the parameter acceptance rate is calculated here as the average of all samples contained within discretised boxes. The quantisation used for plotting each parameter is as follows: Δlog(α) = 0.3, Δlog(β^{pre}) = 0.1, Δlog(β^{post}) = 0.1, Δlog(τ) = 0.55, Δδ = 3.

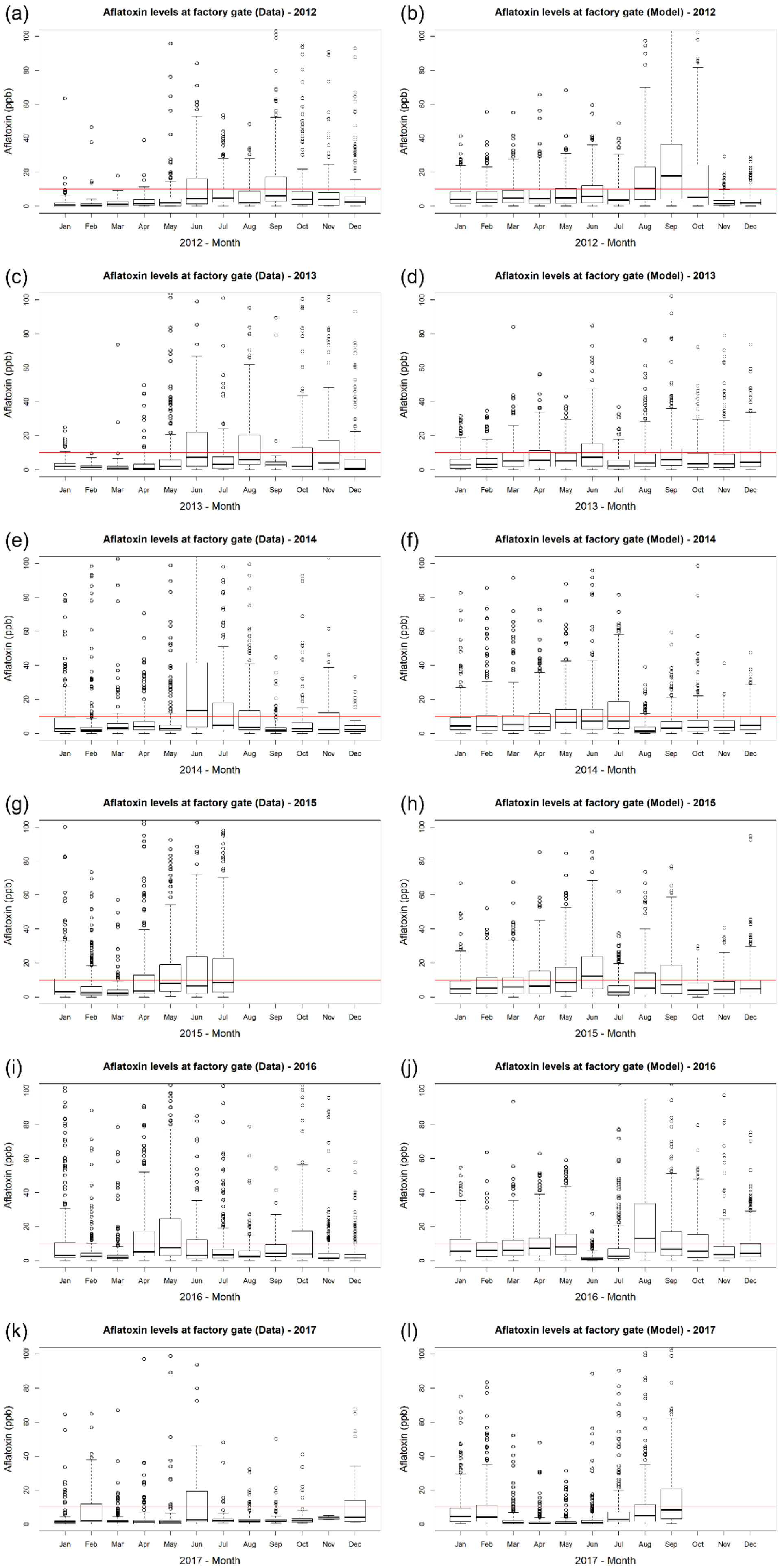
(a)
Aflatoxin levels at factory gate (Data) - 2012
(b)
Aflatoxin levels at factory gate (Model) - 2012
(c)
Aflatoxin levels at factory gate (Data) - 2013
(d)
Aflatoxin levels at factory gate (Model) - 2013
(e)
Aflatoxin levels at factory gate (Data) - 2014
(f)
Aflatoxin levels at factory gate (Model) - 2014
(g)
Aflatoxin levels at factory gate (Data) - 2015
(h)
Aflatoxin levels at factory gate (Model) - 2015
(i)
Aflatoxin levels at factory gate (Data) - 2016
(j)
Aflatoxin levels at factory gate (Model) - 2016
(k)
Aflatoxin levels at factory gate (Data) - 2017
(l)
Aflatoxin levels at factory gate (Model) - 2017
Aflatoxin (ppb)
0
20
40
60
80
100
Jan
Feb
Mar
Apr
May
Jun
Jul
Aug
Sep
Oct
Nov
Dec
2012 - Month
2013 - Month
2014 - Month
2015 - Month
2016 - Month
2017 - Month

Supplementary Figure 2 Aflatoxin boxplots comparing monthly distributions of historical aflatoxin observations at the factory gate and model predicted monthly aflatoxin distributions for all 2012-2017.The red horizontal line indicates the 10-ppb rejection threshold. Outlier values are shown as individual points beyond the 2.5 IQR whiskers.

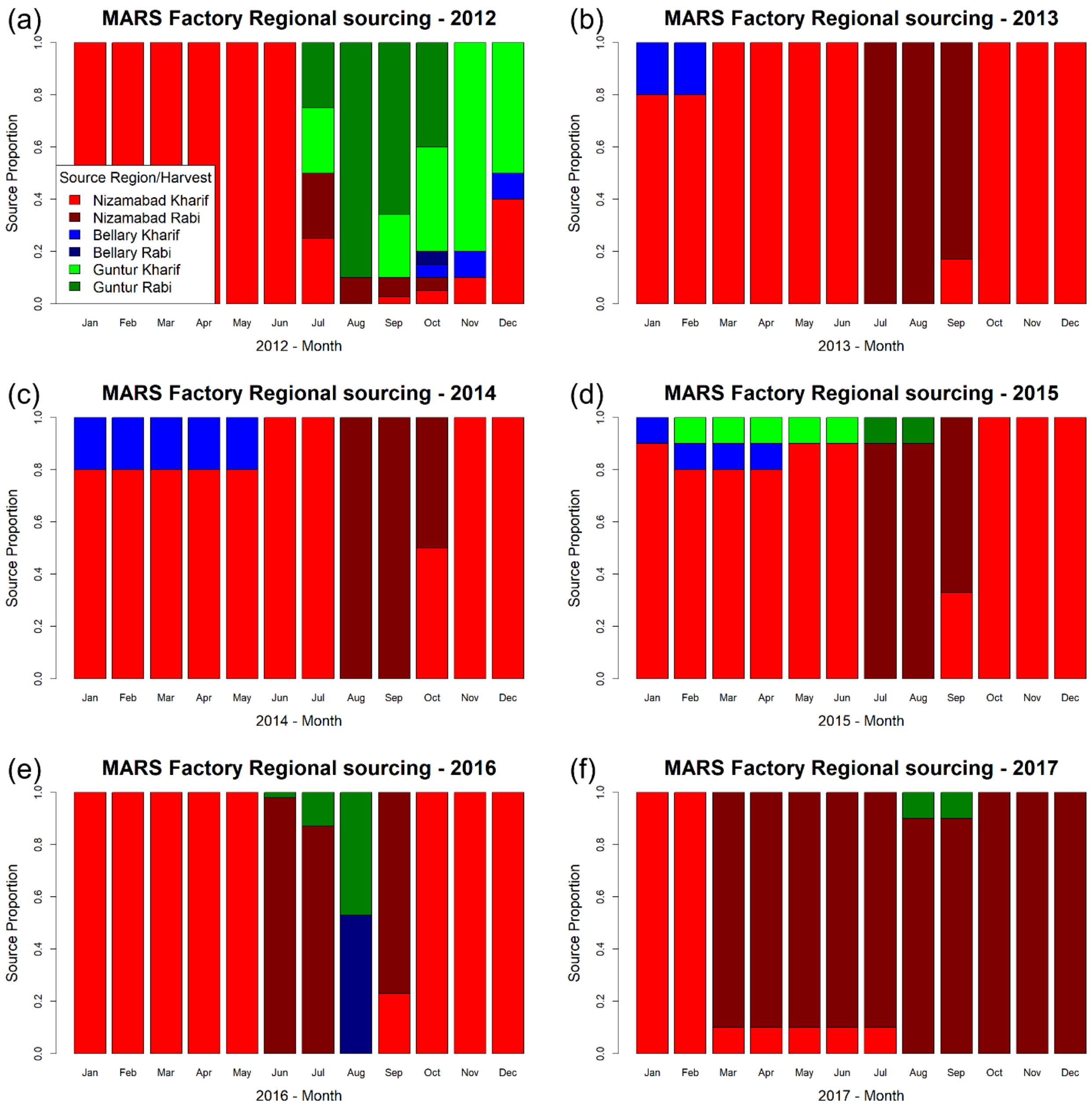

Supplementary Figure 3 Historical monthly maize sourcing profiles for 2012-2017.

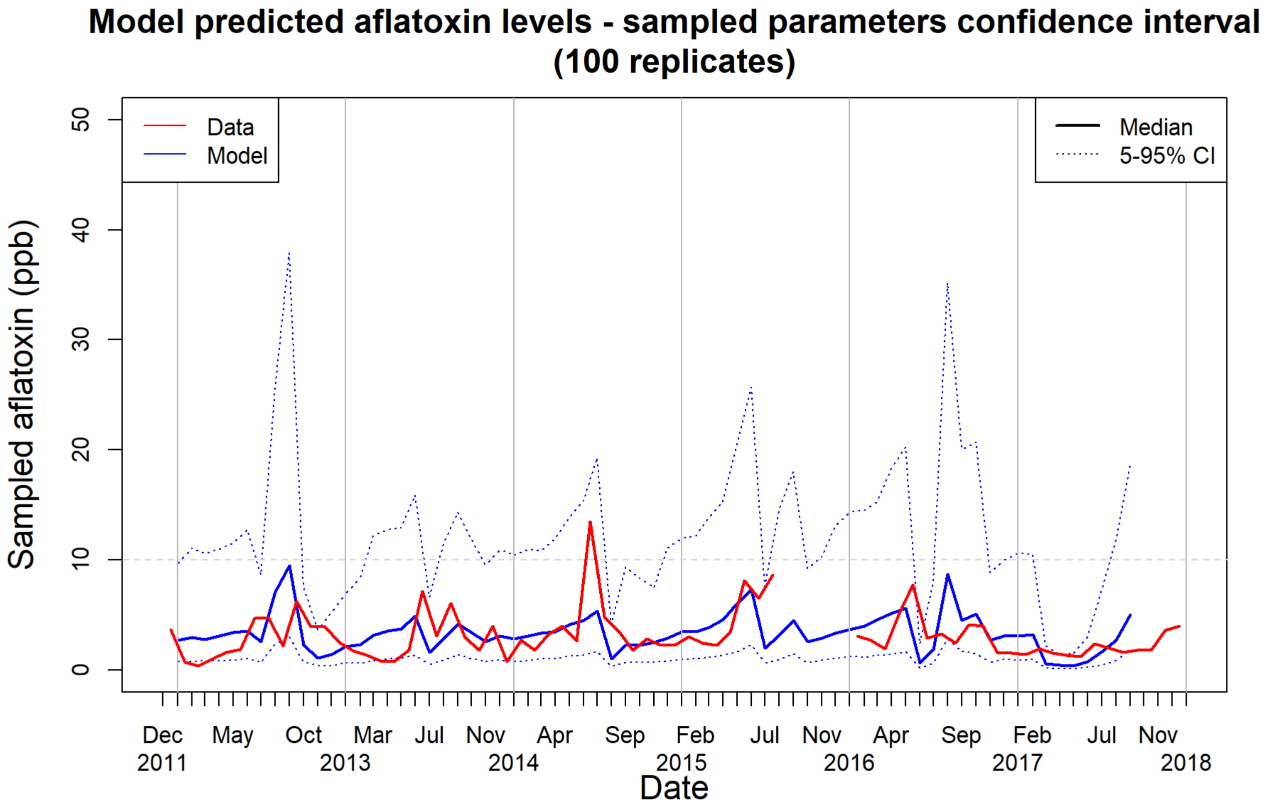

Supplementary Figure 4 Model median confidence interval obtained from 100 independent replicates sampling parameters from the posterior distribution. The red line represents the median of the monthly historical aflatoxin values. The solid blue line represents the median of the hundred model replicate median aflatoxin values for each month, and the lower and upper dotted blue lines represent the bounds of the 5th and 95th percentile of model median monthly aflatoxin values, respectively.

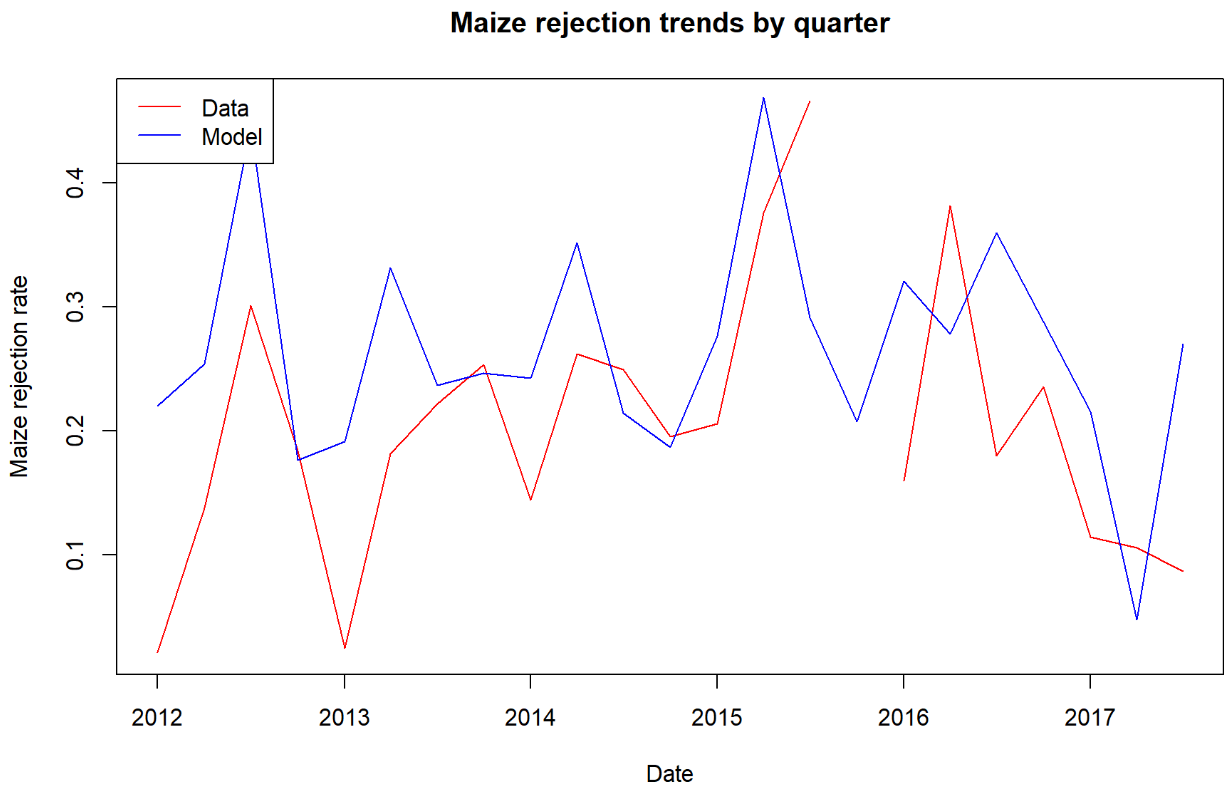

Supplementary Figure 5 Maize rejection rates aggregated on a quarterly interval for the model (blue) and historical data (red).

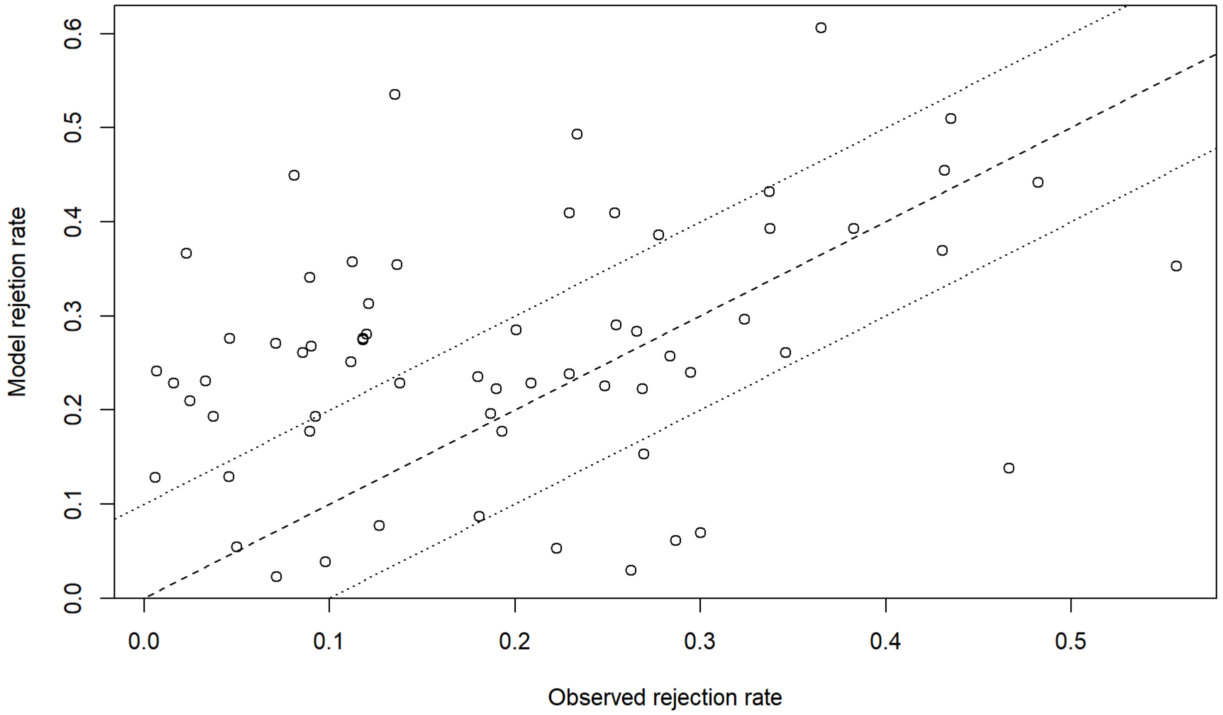

Supplementary Figure 6 Monthly rejection rates scatterplot. The rejection rates for model and observed data for a given month are shown as individual circles for each month. The dashed line denotes where model monthly rejection rate is equal to the historical data monthly rejection rate, and the dotted lines show the +/-10% tolerances used to classify the model results for descriptive statistics.

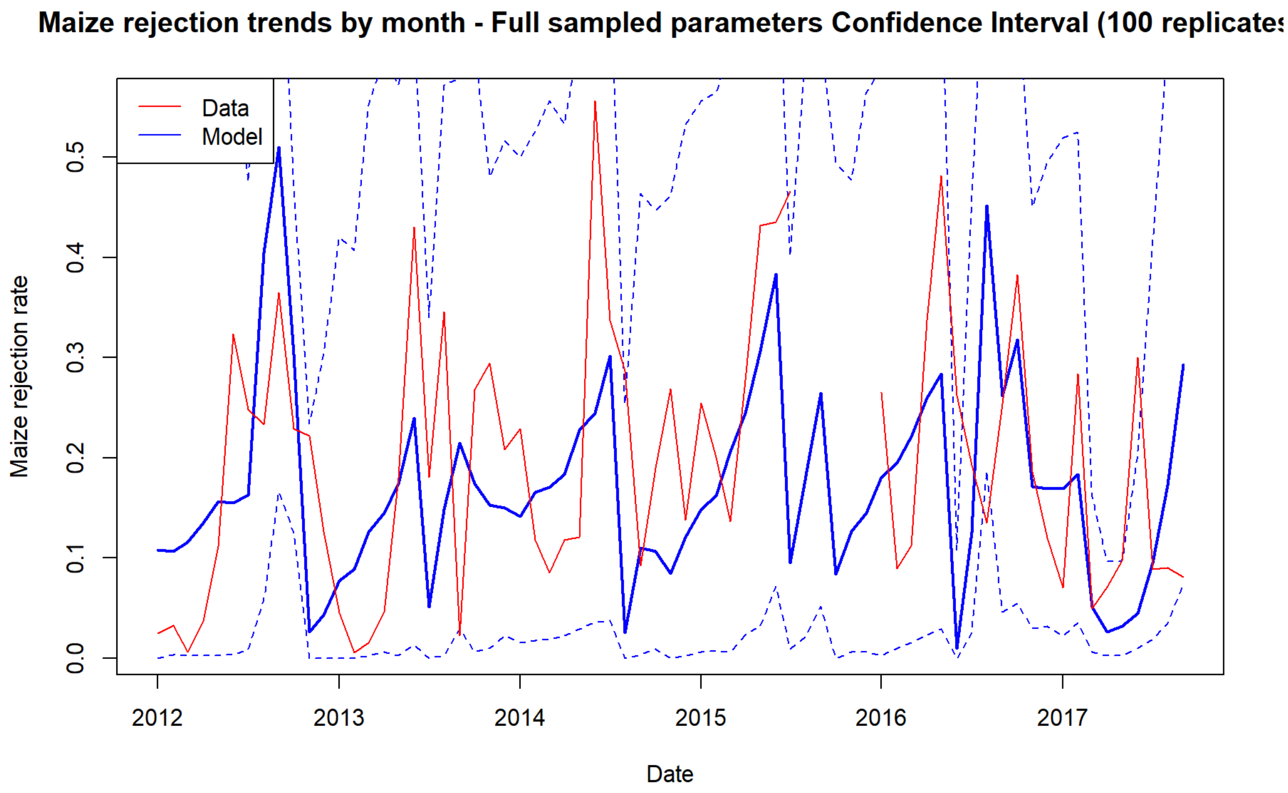

Supplementary Figure 7 Confidence intervals on model predicted rejection rates generated from an ensemble of 100 independent model realisations, sampling from the posterior parameter distribution. The confidence interval is generated for each month by taking the 5th and 95th percentile of model rejection rates for that month from an ensemble of 100 model replicates.

(a)

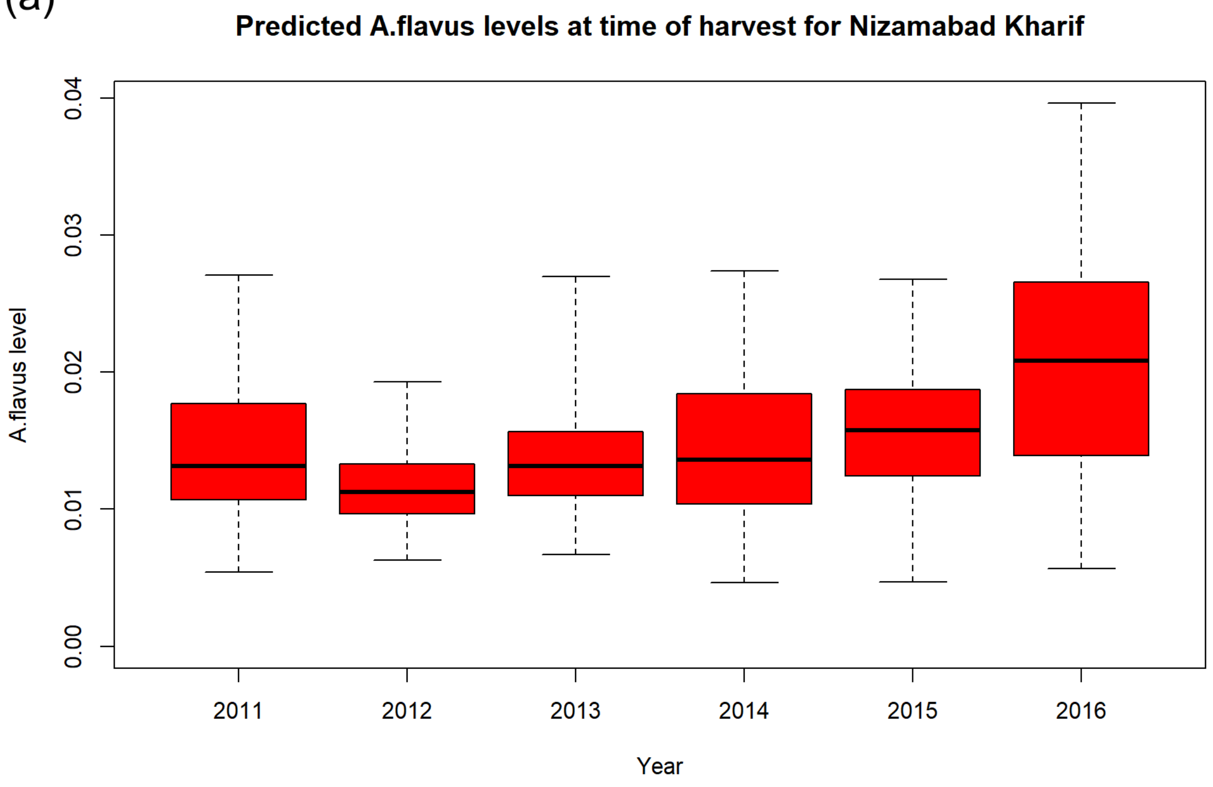

(b)

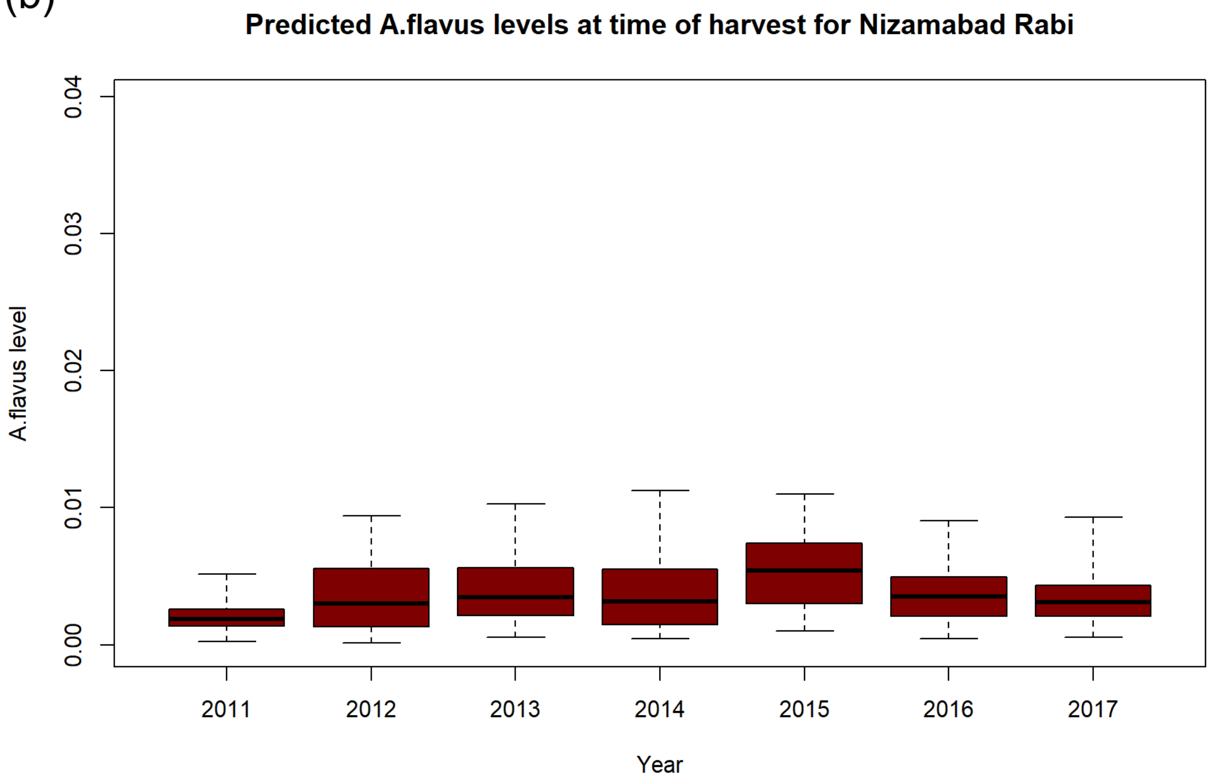

Supplementary Figure 8 Model predicted A. flavus *levels in batches at time of harvest for the (a) Kharif and (b) Rabi cropping seasons in the Nizamabad region from 2011-2017.*

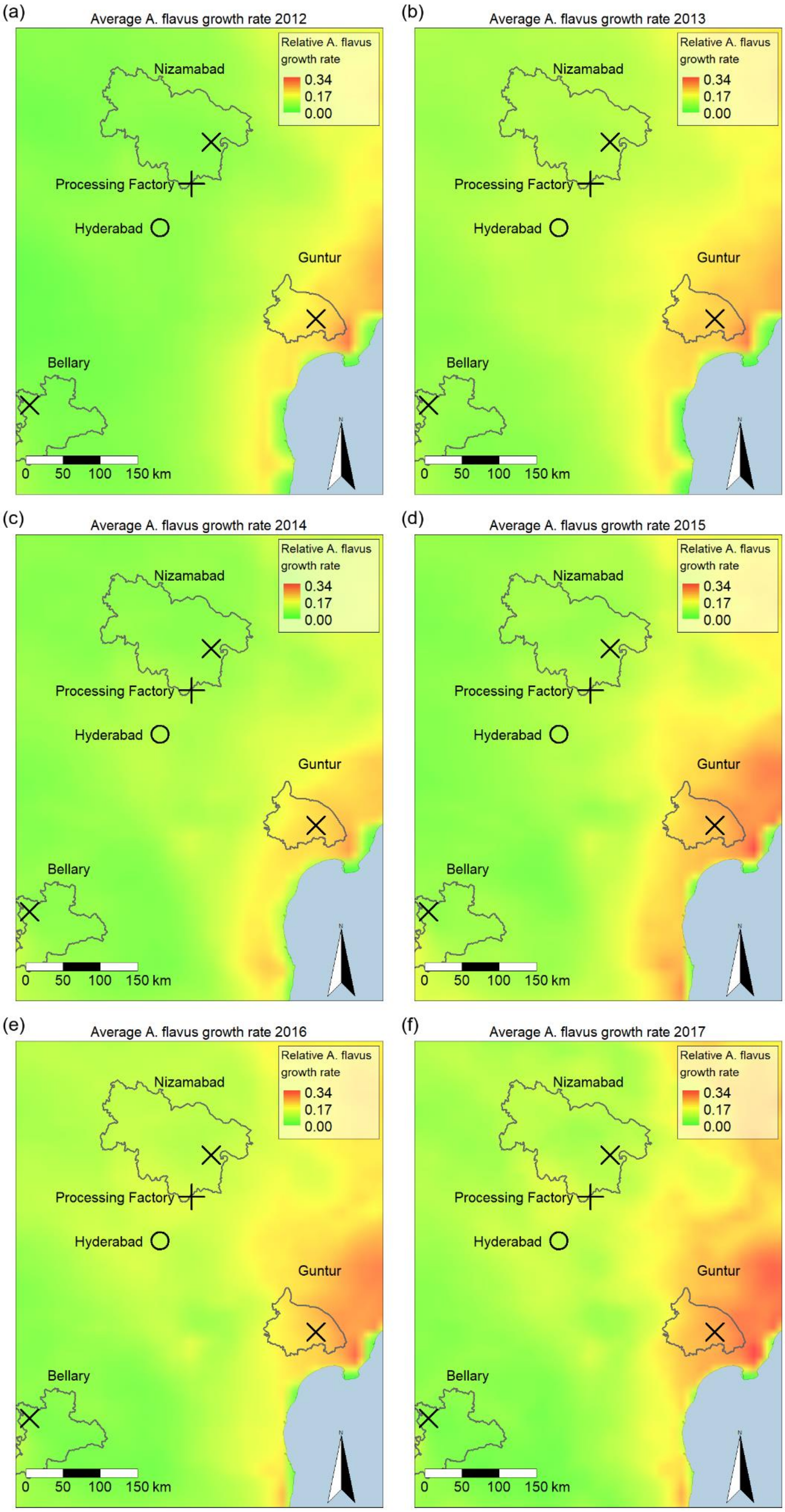

Supplementary Figure 9 Model predicted annual average relative A. flavus *growth rates for 2012-2017. Spatial map averaging the hourly growth rate of* A. flavus *over each year.*

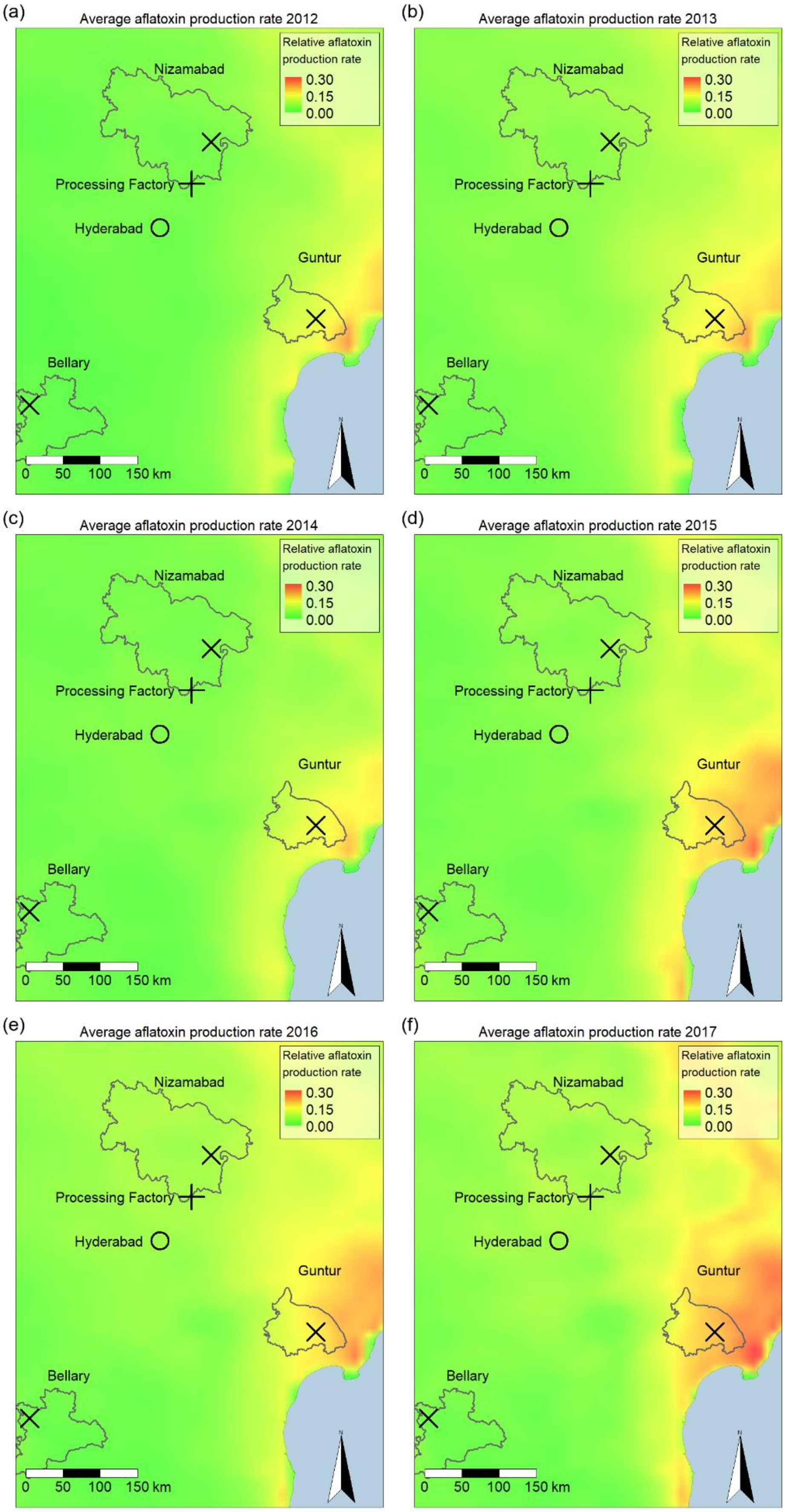

Supplementary Figure 10 Model predicted annual average relative aflatoxin production rates for 2012-2017. Spatial map averaging the hourly production rate of aflatoxin over each year

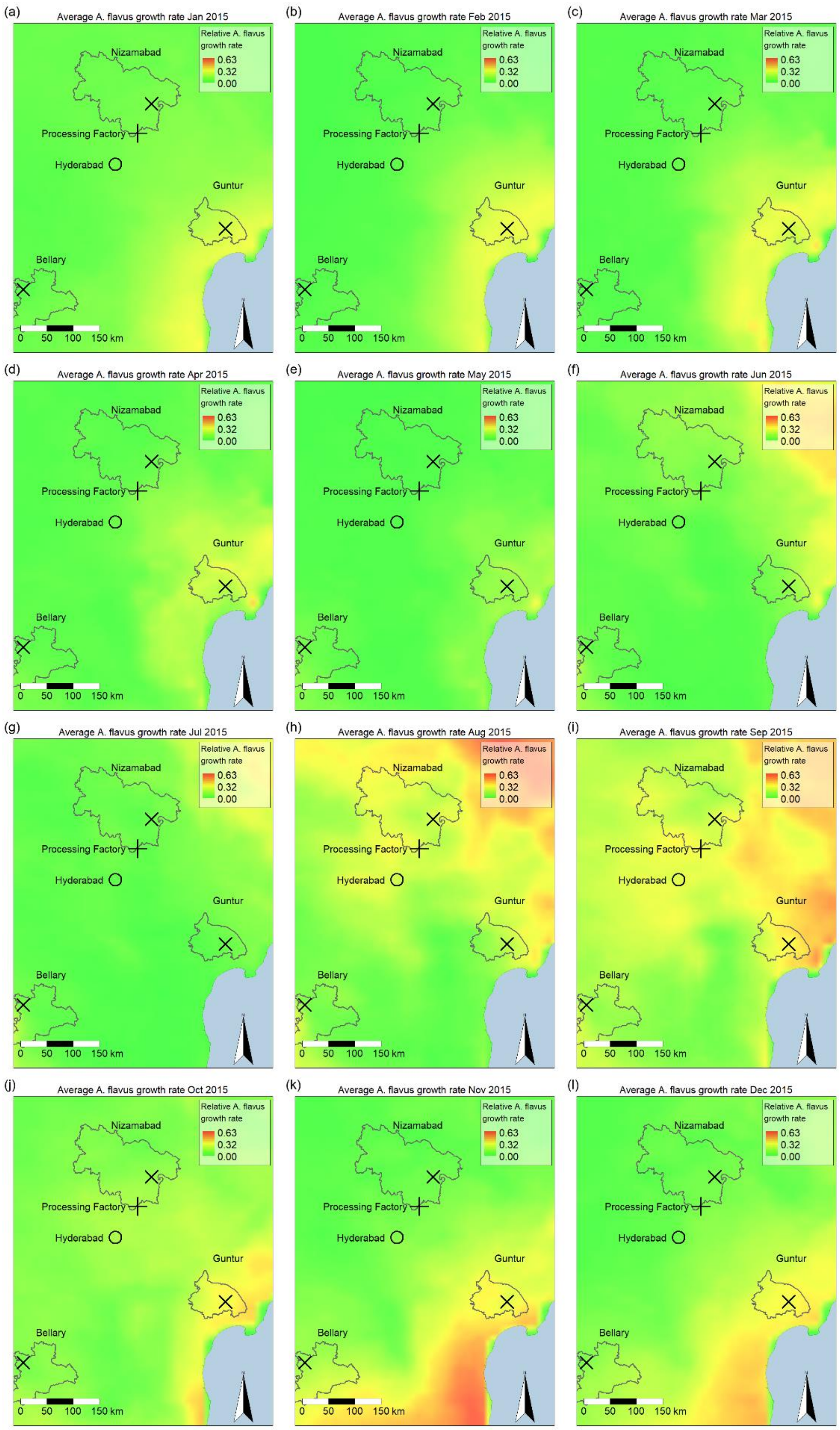

Supplementary Figure 11 Model predicted monthly average relative A. flavus *growth rates for all months in 2015. Spatial maps averaging the hourly growth rate of* A. flavus *over each month in 2015.*

Supplementary Discussion

Aflatoxin boxplots comparing monthly distributions of historical aflatoxin observations at the factory gate and model predicted monthly aflatoxin distributions

The monthly historical and model aflatoxin distributions shown in Supplementary Figure 2 show the correspondence between the model predictions and the historical data. It is notable that the distributions of both historical data and model outputs are highly skewed, with aflatoxin values of many outlier datapoints orders of magnitude higher than median aflatoxin values.

Historical monthly maize sourcing profiles for 2012-2017

Historical data presented in Supplementary Figure 3 indicates that most material was sourced for the factory from the Nizamabad area. The larger Kharif crop is also more commonly used for sourcing, with the Rabi crop typically being sourced in the mid-late year periods. When Nizamabad is not the sole sourcing region, material may be obtained from Bellary, typically in the first five months of the year as in 2013-2015, or Guntur towards the middle of the year. Sourcing in 2012 was more diverse than in later years, anecdotally the switch to more focused sourcing profiles was driven by high observed contamination levels that year.

Model median confidence interval

A confidence interval for the model predictions, subject to uncertainty in the estimated parameters and model stochasticity is presented in Supplementary Figure 4. An ensemble of 100 model replicates is generated, each using an independent sample from the posterior parameter distribution. From this ensemble we select the median monthly aflatoxin timeseries and construct the median confidence interval as the interval that at each month is above the lowest 5% of model medians and below the top 95% of model medians. The “median of medians” is similarly constructed as the line that for each month lies at the median of all ensemble median values. We note that the “median of medians” presented here represents a significantly smoothed quantity compared to the median of any individual realisations, and thus the shape should be interpreted in terms of general scale, rather than month-to-month variations. We note that the historically observed median is entirely contained within the confidence interval of the model predictions, showing that over multiple realisations the model can achieve all features present in the historical data.

Modelling rejection trends at the processing factory: additional details

Further examination of rejection rates in Supplementary Figure 5 and Supplementary Figure 6 demonstrates that while the model may overestimate rejection rates on shorter timescales, the overall rejection rates are more similar to historical data when averaged over longer time periods. This effect may in part be due to the uncertainty in sourcing origin being smoothed in the quarterly aggregation. As evident in Supplementary Figure 6 the model is predominantly overestimating, particularly in times when the historical rejection rate was low (the upper-left quadrant of the plot). Conversely, there are very few times with a high historical rejection rate not captured by the model.

Supplementary Figure 7 presents the confidence interval for the model predicted rejection rates subject to uncertainty in model parameters and model stochasticity. This confidence interval for the rejection rates is constructed analogously to the median aflatoxin confidence interval presented in Supplementary Figure 4. Again, we see that the full confidence interval bounds the historical rejection rate distribution. We also note again that the median of medians for the rejection rate averages out month to month variability and so the focus should be on the scale, rather than month-to-month variability.

Inter-year variability in model predictions of A. flavus levels for Kharif and Rabi crops in one sourcing region

To provide general insight and understanding of the system, the model can be used to investigate general trends within and between regions and cropping seasons. As Supplementary Figure 8 shows, within the Nizamabad region, Rabi harvests are predicted to have significantly lower A. flavus loads than Kharif harvests, demonstrating also significantly lower variability both within and between years. These types of result can allow users of the model to get an overview of the general behaviour of the system and investigate any relationships of interest.

In plots Supplementary Figure 9 and Supplementary Figure 10 we see annual average suitability for 2012-2017 for *A. flavus* and aflatoxin production, respectively. We see broadly similar trends between years for regions of highest risk, however with marked variation in overall risk levels from year to year. Supplementary Figure 11 shows within year suitability for *A. flavus* growth in 2015 as monthly averages, and these again show significant variability from month to month, with more pronounced regional variations than the annual risk maps. Temporally we see August to November has significantly higher risk levels than the rest of the year.

Supplementary References

1. Shaykewich, C. F. An appraisal of cereal crop phenology modelling. *Can. J. Plant Sci.* **75**, 329–341 (1995).
2. Yan, W. & Hunt, L. A. An equation for modelling the temperature response of plants using only the cardinal temperatures. *Ann. Bot.* **84**, 607–614 (1999).
3. Service, M. S. U. C. E. & Project, N. C. H. *National Corn Handbook*. (Cooperative Extension Service, Michigan State University, 1985).
4. Battilani, P., Camardo Leggieri, M., Rossi, V. & Giorni, P. AFLA-maize, a mechanistic model for *Aspergillus flavus* infection and aflatoxin B1 contamination in maize. *Comput. Electron. Agric.* **94**, 38–46 (2013).
5. Li, D.-W. & Kendrick, B. A year-round study on functional relationships of airborne fungi with meteorological factors. *Int. J. Biometeorol.* **39**, 74–80 (1995).
6. Kruit, R. J. W., Pul, W. A. J. van, Jacobs, A. F. G. & Heusinkveld, B. G. Comparison between four methods to estimate leaf wetness duration caused by dew on grassland. in *26th Conference on Agricultural and Forest Meteorology (26AG), Boston, 2004* (American Meteorological Society, 2004).
7. Siriacha, P., Kawashima, K., Kawasugi, S., Saito, M. & Tonboon-Ek, P. Postharvest contamination of Thai corn with *Aspergillus flavus*. *Cereal Chem.* **66**, 445–458 (1989).
8. Sydenham, E. W., van der Westhuizen, L., Stockenström, S., Shephard, G. S. & Thiel, P. G. Fumonisin-contaminated maise: Physical treatment for the partial decontamination of bulk shipments. *Food Addit. Contam.* **11**, 25–32 (1994).
9. Battilani, P., Formenti, S., Ramponi, C. & Rossi, V. Dynamic of water activity in maize hybrids is crucial for fumonisin contamination in kernels. *J. Cereal Sci.* **54**, 467–472 (2011).
10. Chayjan, R. A. & Esna-Ashari, M. Modeling of heat and entropy sorption of maize (cv. Sc704): neural network method. *Res. Agric. Eng.* **56**, 69–76 (2010).
